# There and Back Again: Recovery of Terephthalic Acid from Enzymatically Hydrolyzed Polyesters for Resynthesis

**DOI:** 10.1021/acssusresmgt.4c00430

**Published:** 2025-01-23

**Authors:** Chiara Siracusa, Virginia Celestre, Felice Quartinello, Giacomo Damonte, Jeppe Madsen, Georg M. Guebitz, Anders Egede Daugaard, Alessandro Pellis

**Affiliations:** † acib GmbH, Konrad-Lorenz-Strasse 20, 3430 Tulln an der Donau, Austria; ‡ Institute of Environmental Biotechnology, Department of Agrobiotechnology, IFA-Tulln BOKU University Vienna, Konrad-Lorenz-Strasse 20, 3430 Tulln an der Donau, Austria; § Danish Polymer Centre, Department of Chemical and Biochemical Engineering, Technical University of Denmark, DK-2800 Lyngby, Denmark; ∥ Department of Chemistry and Industrial Chemistry, Università degli Studi di Genova, Via Dodecaneso 31, 16146 Genova, Italy

**Keywords:** Enzymatic depolymerization, monomer recovery, chemical resynthesis, bio-chemical plastic recycling, closure of the carbon loop, cutinases

## Abstract

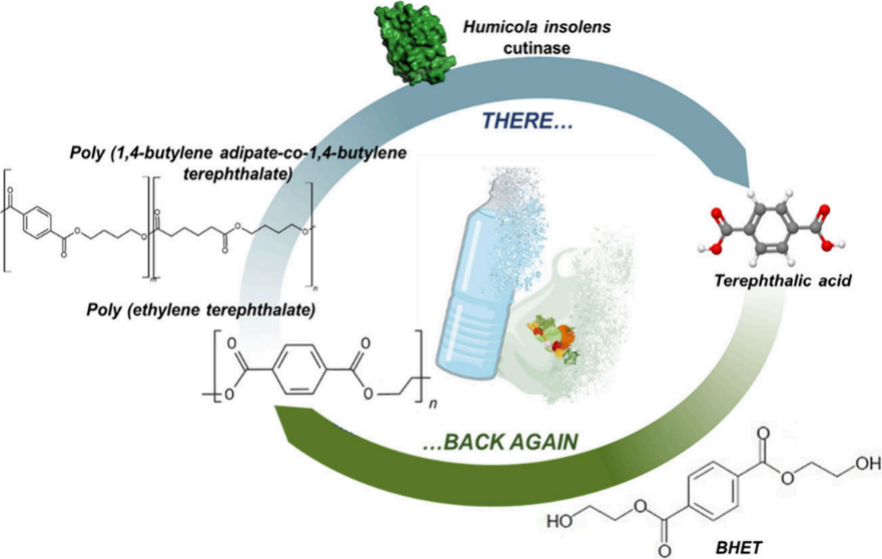

Poly(ethylene terephthalate) (PET) is still a major player in the plastics industry, especially for packaging. Despite attempts to derive its basic components from biological resources, production of terephthalic acid (TPA), one of the two PET monomers, still depends on fossil resources. Alongside traditional polyesters, TPA is a building block also for biodegradable polymers, such as poly(1,4-butylene adipate-*co*-1,4-butylene terephthalate) (PBAT). Here, PET, PBAT, and real plastic waste were successfully depolymerized using *Humicola insolens* cutinase as an environmentally friendly alternative to mechanical or chemical treatments allowing recovery of TPA even from mixed plastic waste. This monomer was isolated in high purity upon acidification as confirmed by using Fourier Transform-Infrared Spectroscopy, ^1^H-NMR spectroscopy, and Thermogravimetric analysis. Consequently, contaminants or residual buffer salts caused major issues during synthesis of PET precursors upon reaction with ethylene glycol (EG) and TPA. The recovered TPA was used to prepare bis(hydroxyethyl) terephthalate (BHET) and further repolymerized to PET. The resulting molecular weight of the polyesters was found to be dependent on the purity of the TPA and on the catalyst used.

## Abstract 2

This work describes the enzymatic depolymerization of two different polyesters (poly(ethylene terephthalate) and poly(1,4-butylene adipate-*co*-1,4-butylene terephthalate)) and the subsequent recovery of terephthalic acid in a suitable purity for resynthesis of PET.

## Introduction

The demand for plastic products is expected to dramatically increase, as reported in the most recent revision of the United Nations (UN) environment program assessing plastic pollution.^1^ Together with the European Green Deal,^2^ it encourages a decrease in production that must be coupled with novel competitive strategies for improving the plastic’s recycling rates. Current efforts are addressing better quality polymer formulations with good durability and applicability, proving to be simultaneously optimal for the implementation of after-life recycling or upcycling strategies. The proposals include the establishment of a mandatory percentage of recycled material that can be incorporated into newly manufactured products. Nevertheless, plastics are still mostly downcycled,^3^ since the quality of the recycled material is in almost the totality of cases lower than the original material, as a consequence of unintended additives originating from the life-cycle and handling of the materials.

Biodegradation has been explored since the 1990s^4^ as a mild, environmentally-friendly alternative for recycling plastic waste. The approach is based on the observation that, in a natural environment (either in soil or in aquatic environment), plastics could be colonized by microorganisms, which secrete enzymes with hydrolytic activity able to partially depolymerize the polymers.^5^ This phenomenon can be exploited in a controlled environment to specifically recover elementary building blocks, coupling waste management with high-quality, sustainable recycling.

The first demonstration of the effective enzymatic treatment of PET was provided by Müller et al.^6^ in 2005. Since more than ten years ago, these findings prompted a deep resonance through omics technologies and enzyme engineering, leading to the identification and improvement of several enzymes active on polyesters, either for surface modification or bulk depolymerization.^7^ As an example, some cutinases that yield outstanding PET hydrolysis rates have been recently characterized from metagenomic analysis of compost material: leaf-branch compost cutinase (LCC)^8^ and polyester hydrolase 7 (Phl7).^9^ Nowadays, protein engineering aims at optimizing these novel catalysts for the heterogenicity of available substrates and the future application in large-scale processing facilities.^10^

The evergreen prominence of PET in the plastics industry (58.7 Mt yearly demand in Europe^11^) justifies its prioritization in recycling studies. PET has a wide variety of applications ranging from water and soft drink packaging to textile industry products. This contributed to make it the most mechanically recycled plastic per volume.^12^ Its depolymerization results in the release of ethylene glycol and terephthalic acid (TPA). Beside PET, TPA-based polymers include other versatile polyesters such as poly(1,4-butylene adipate-*co*-1,4-butylene terephthalate) (PBAT), largely combined with starch and other polymers for food packaging applications, and poly(trimethylene terephthalate) (PTT)^13^ commonly used for carpet fibers. The addition of aliphatic moieties tunes the mechanical properties and improves the overall susceptibility to hydrolysis.^14^

TPA, as the essential building block for these polymers, is mostly obtained from *para*-xylene (*p*X) via aerobic catalytic oxidation.^15,16^ Different studies have made significant efforts towards the production of biobased TPA,^16,17^ in particular looking at the biobased pX route,^18^ but at the moment, several techno-economic challenges hinder the implementation of these processes at an industrial level. Recovering TPA from hydrolyzed material and performing the resynthesis of PET would close the loop and significantly reduce the dependence of petrochemical raw materials for production of TPA containing polymers.

The purification and recycling of TPA has been achieved for PET through a NaOH-based hydrolysis and with the aid of organic solvents, as reported by Lee et al.^15^ Other works focus on the purification of the residual monomers in solution, such as ethylene glycol for PET recovered through distillation and membrane separation,^19^ as well as adipic acid and butanediol which were successfully separated through nanofiltration.^20^ Nonetheless, despite a large number of studies focusing on enzymatic depolymerization of PET,^21^ very little research has investigated the monomer recovery^22^ and even less coupled with resynthesis application. Similarly, PBAT degradation has been proven^23^ and also chemically based depolymerization with a focus on separation of its monomers.^24^ Nevertheless, the possibility of extending the same approach of TPA derivation from PET also to different substrates such as PBAT hydrolysates or real mixed waste containing PET, all enzymatically treated, remains of high interest for versatile convenient upcycling. Therefore, this study investigates the enzymatic depolymerization of various TPA-based polymers (PET, PBAT) and of PET rich mixed plastic waste as possible sources for the recovery of this aromatic diacid. Particular attention was given to the reintegration of TPA into the polymer synthesis process. The final objective was to demonstrate the possibility of overcoming the traditional, linear mechanical recycling, heading toward a completely circular approach with a particular focus on using the isolated TPA directly for repolymerization without additional purification steps.

## Results and Discussion

### Hydrolysis of PET and PBAT

A commercially available enzyme, namely *Humicola insolens* cutinase, was chosen as well-described for the hydrolysis of synthetic polyesters.^25^ Yet, there is a lot of future potential for optimization of the hydrolysis step due to a number of more efficient PET-hydrolyzing enzymes developed over the last decade by our group and others.^8,9,26^ The enzyme formulation had a total protein concentration of 9.8 mg mL^–1^ and an activity of 168.78 U mg^–1^ on *p*-NPB. The incubation temperature was adjusted at 70 °C as a compromise between the thermal stability of the enzyme and the Glass Transition Temperature (*T*_g_) of the polymers (∼70 °C for PET). To be noted that for enzymatic recycling for cellulose-based textiles, only less than 5% activity loss after reusing the enzyme in five cycles^27^ was observed. Moreover, the cutinase used in this study is also used for large-scale industrial applications such as stickies control in paper mills and is hence commercially available in bulk quantities.^28^ Considering other industrial applications of enzymes, it is well known that for example cellulases are used for industrial enzyme treatment on hardwood kraft-based dissolving pulp^29^ or also for ethanol production, where it has been proven to be recyclable^30^ therefore opening interesting perspectives for enzyme recycling also in the proposed technology.

Various pure PET specimens as well as PBAT specimens were hydrolyzed. cPET did not undergo hydrolysis under the applied conditions (Table S3, SI). A first incubation with films sized 0.5 cm × 3 cm proved the susceptibility of PET1 and PET3, as well as both PBAT specimens to enzymatic attack (Figures S2–S3). Films sized 10 cm × 3 cm were incubated in larger volume but under identical conditions and enzyme concentrations. Weight losses of 51% and 14% were recorded for PET1 and PET3 films (Figure S2B) after 72 h of incubation. While DSC indicated that both PET1 and PET3 were amorphous (Figure S4 and Table S4 in the SI), a lower thickness of the PET1 film was most likely beneficial for hydrolysis (250 μm PET3 vs 100 μm PET1). For PET2, DSC revealed a high crystallinity of around 35% which in agreement with previous reports^31^ well explained the recalcitrance of the sample to the enzymatic attack. On the other hand, with an increase in the enzyme concentration from 5 to 15 μM HiC, PET1 was almost completely (96%) degraded within the same time interval (Table S5, SI). When compared to PET, expectedly PBAT1 and PBAT2 were hydrolyzed much faster and were completely degraded within 6 and 24 h of incubation.

Two types of real waste samples containing micronized PET (PW1 and PW2) were also enzymatically treated to study the recovery of TPA under these conditions. Both samples were susceptible to enzymatic hydrolysis and led to weight losses of 56% and 51% for PW2 and PW1, respectively, after 72 h of incubation. At the final time point of the incubation, TPA concentrations of 51.6 mM TPA (PW2) and 42.2 mM TPA (PW1) were measured through HPLC. For the more crystalline sample, a slight increase of crystallinity from 17% to 22% was seen according to DSC (Figure S5, Table S6 in the SI) indicating preferential hydrolysis of amorphous regions in agreement with previous reports.^31^ This effect was not seen for the less crystalline (11%) waste sample.

### Quantification of the Released Soluble Products

Released products were normalized with the initial weight and plotted in milligrams per milligram of polymer, allowing a direct comparison of the efficiency of depolymerization into monomers of each sample. Ethylene glycol (EG) and terephthalic acid (TPA) are the monomers derived from PET depolymerization, while adipic acid (AA) and 1,4-butanediol (BDO) are the remaining building blocks of PBAT.

As visible in Figure S3(A,D) panels, the released monomers consistently increased with time. Utilizing TPA as a reference for monomers (Figure S3, gray bars), from PBAT1, TPA was released incrementally from 0.05 mg (6 h) to 0.22 mg (72 h) per mg of original polymer (Figure S3A, gray bar). Slightly higher amounts were measured for PBAT2 hydrolysates (Figure S3B, gray bar). Both formulations of PET resulted in similar trends. PET1 and PET3 released TPA respectively for 0.21 and 0.15 mg per mg of polymer after 72 h of incubation (Figure S3C–D, gray bar).

PBAT and PET hydrolysis was performed in 50 mL total volume to determine whether the enzyme still performs with a larger amount of initial material and investigate the possibility of monomer recovery.

These reactions with a 20 times higher amount of incubated plastic films gave similar trends in terms of released monomers in solution, as shown in Figure S6(A-D panels). The TPA concentration increased progressively with time for all the samples, while the aliphatic monomers of PBAT were recorded with minor differences between the two PBAT samples: confirming the findings using sample sizes of 0.5 × 3 cm. Changes in polyester film surfaces were analyzed through FT-IR and reported in the SI (Figures S7-S10, Table S7).

### TPA Recovery

After hydrolysis, soluble TPA in the hydrolysate was quantified via HPLC analysis. As described in previous work,^32^ acidification of the solution was meant to decrease the solubility of those monomers with a higher pKa than the final pH, triggering monomer precipitation. The monomers were then recovered, purified, and dried for characterization and resynthesis. Figure S11 and Table S8 show the recovery and monomer concentration before and after HCl mediated precipitation.

**Figure 1:**
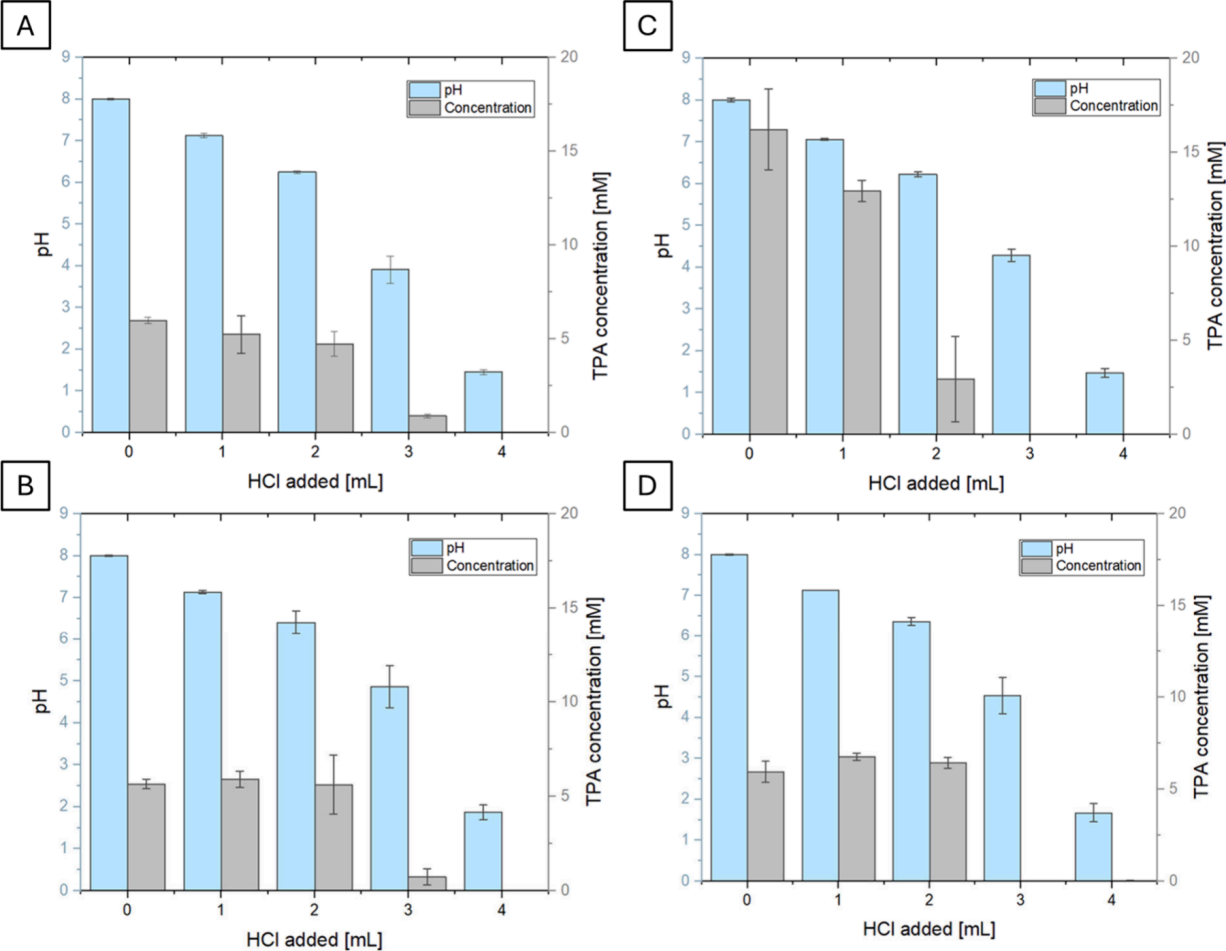
Progression of pH (light blue bars) assessed through a pH probe and TPA concentration (gray bars) measured through HPLC with the sequential addition of 6 N HCl (from 1 to 4 mL). A: PBAT1; B: PBAT2, C: PET1; D: PET3. The represented data are the average of triplicate measurements.

In a first step, recovery of TPA was studied with the pure samples since contaminants of the waste sample may influence the process. In all the cases, a progressive decrease in pH was accompanied by a drop in TPA concentration: by way of explanation, PBAT1 TPA concentration varied from initial 5.2 mM to 5 mM after 1 mL of 6 HCl addition, to an undetectable amount after 4 mL of 6 N HCl addition (Figure 1A). Similar trends were observed in the other hydrolysates, both PET and PBAT samples, where after the addition of 4 mL of HCl, no residual TPA was detected in the supernatant, even with a higher starting TPA concentration (Figure 1B-D).

Expectedly, it was possible to recover a higher amount of TPA from PET1 than PET3, consistent with the larger weight loss, and the HPLC-determined monomer concentration. PET3 had a lower hydrolysis yield (5.94 mM soluble after 72 h of reaction compared to PET1 16.21 mM), and therefore less TPA could be recovered (112.5 mg from the former, 218.5 mg of TPA from the latter, which corresponds for both samples to >95% recoverable soluble TPA). Similar observations were done for both formulations of PBAT where 30 mg and 61.5 mg of TPA (corresponding to 65% and >95% yield) could be recovered in order from PBAT1 and PBAT2.

Using an identical protocol, the recovery of TPA from waste samples was studied. A progressive addition of 6 N HCl led to complete TPA precipitation as no TPA was detected in the supernatant via HPLC. The method for TPA recovery proved suitable as well in the case of real mixed plastic waste and allowed the recovery of ∼8 and ∼11 g of TPA respectively from PW1 and PW2 (corresponding to a yield of 57% and 65% of the recoverable soluble TPA in each solution). The presence of additional materials, including pigments, together with the larger amount of incubated material and recovered pelleted TPA after centrifugation partially explains the inferior yield of recovery, suggesting the need for future improvements of the final purification step for larger volumes.

The residual materials were analyzed by DSC revealing some additional melting peaks, which were attributed to the possible presence of polyolefins in these mixtures (see Figure S4). In particular, the thermogram of the treated PW1 sample, PW1-residual, showed the presence of a small melting peak at 130 °C, which was hypothesized to be linked to the presence of HDPE.^33^ In contrast, the DSC curve of the treated PW2 sample, PW2-residual, showed two different peaks at 110 °C and 160 °C, that were consistent with the possible presence of LDPE and PP respectively.^34,35^ Since polyolefins were obviously present only in minor amounts, these peaks were only detected after the enzymatic removal of PET.

### Analysis of Recovered Materials

After recovery, purification, and drying, each sample was analyzed through FT-IR, ^1^H-NMR, and TGA to evaluate the TPA purity, assessing the recovery yield and the material suitability for PET resynthesis. FT-IR and ^1^H-NMR results are discussed in the SI (Figures S12-S13).

**Figure 2:**
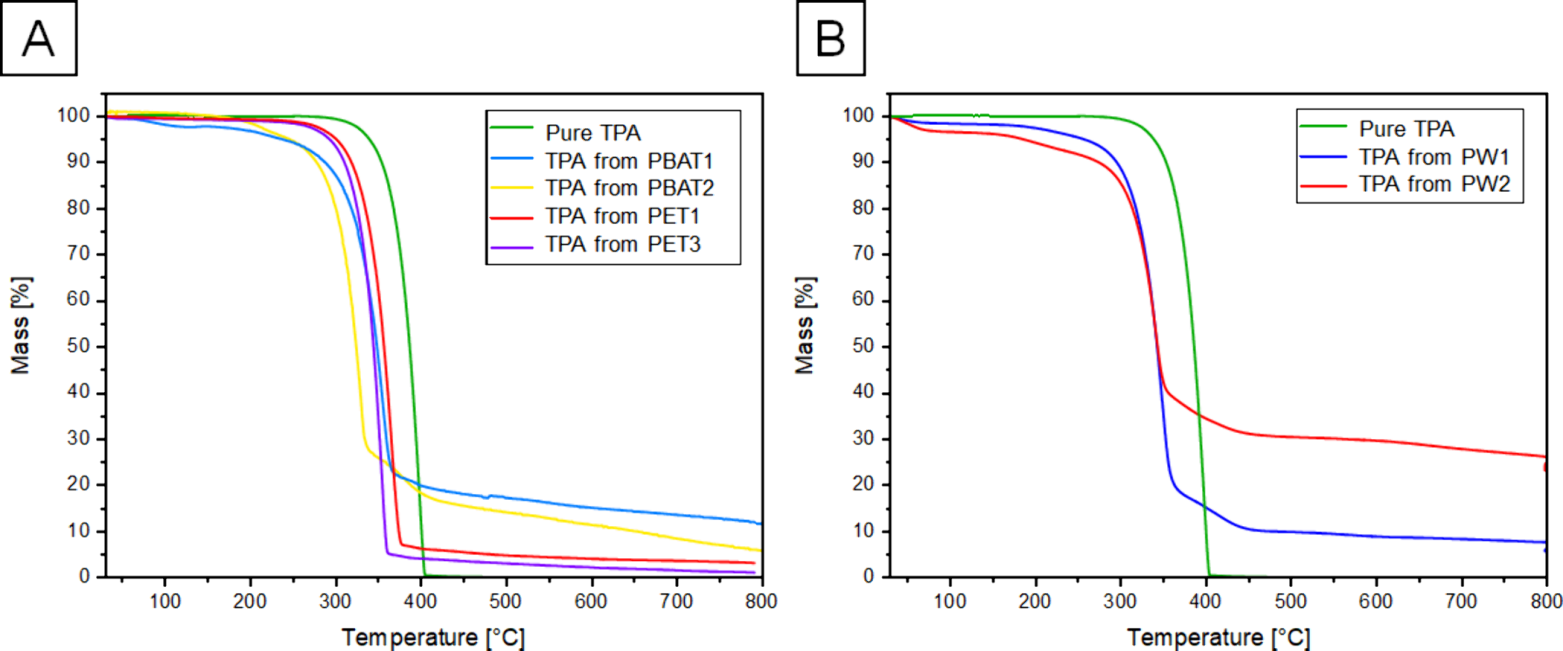
A: TGA thermograms of TPA recovered from 10 × 3 cm film hydrolysis were compared to reference pure TPA. B: TGA plots of TPA recovered from PW1 and PW2 hydrolysates were compared to the reference pure TPA.

The thermal behavior of each recovered TPA sample was analyzed by TGA and estimated in comparison with the pure commercial chemical. TPA recovered from PET1 and PET3 shows a high purity in their diacid form, having a residual mass of 3% and 1% at 800 °C, while the samples resulting from PBAT hydrolysis after combustion recorded a residue of 22% from PBAT2 and ∼9% from PBAT1 (Figure 2A).

Regarding mixed waste materials, TGA leaves a 22% residual material from the PW2 derived sample and only ∼5% from PW1, confirming a higher similarity to thermal behavior of pure TPA (Figure 2B). Additional details are given in Table S12, SI.

The residual material in the TGA along with the absence of additional peaks in the ^1^H-NMR indicates that a fraction of the material remains in the salt form. In particular, alkali hydrogen terephthalates have been shown to exhibit such complex weight loss curves.^36^ This is consistent with a lower apparent purity, as determined by Q-NMR (see Figure S22 and Table S13).

### Resynthesis of TPA to BHET and Oligo(ethylene terephthalate)s

**Scheme 1:**
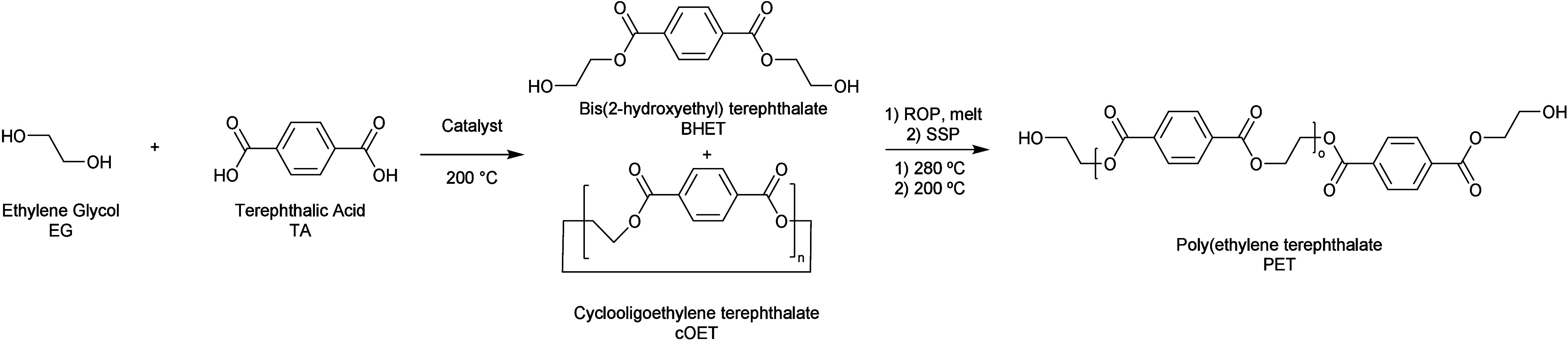
Reaction between Terephthalic Acid and Ethylene Glycol to Form Monomers for PET Polymerization, Followed by Ring-Opening and Condensation Polymerization to Form PET with 2-Hydroxyethyl End-Groups

Industrially, the synthesis of PET is commonly accomplished through terephthalic esters rather than directly from the acid.^37^ To investigate an industrially relevant process, the process pursued involved first preparing a diester of TPA, bis(hydroxyethyl) terephthalate, by reacting the crude acid with two equiv of EG. The possibility of polymerizing the resulting product was then investigated. The process is outlined in Scheme 1.

#### Reaction between EG and TPA

##### Stoichiometry, Initial Trials with Titanium(IV) Butoxide

Initial trials conducted on a 50 mg scale in standard 5 mm NMR tubes revealed that bulk mixtures of commercial high-purity grades of EG and TPA in the presence of a catalytic amount of titanium(IV) butoxide formed BHET with a fraction of more than 70% in a stoichiometric ratio of 2:1 (Figure S23). Higher amounts of EG increased TPA dissolution and relative BHET content. Thus, a more homogenous reaction mixture along with an excess of alcohol is believed to contribute to the higher yield. However, to maintain a good atom economy, a 2:1 ratio was kept, despite lower overall yield. As illustrated in Figure S24, the reaction also produced cyclic oligoethylene terephthalates, ((c)OET) (see Scheme 1 and Figure S23). Based on the literature, these are likely to be a mixture of linear and cyclic oligomers^38,39^ which are denoted as (c)OET here. Any linear OET can participate in the polycondensation reaction along with BHET, and in principle, cyclic OET ((c)OET) can directly form linear PET through ring-opening polymerization.^40^ Therefore, the presence of these species is not expected to negatively affect PET synthesis. When evaluating the stoichiometry between EG and TPA, the purity of the recovered TPA was considered to be 100%, to keep the mass concentration constant. In fact, Q-NMR showed that the actual amount of TPA was found to be lower (see above). As a result, the *de facto* stoichiometry is larger than 2 for the recovered TPAs.

##### Catalyst, Pure Samples

Two different catalysts were applied in the reaction between TPA and EG: the “green” titanium(IV)butoxide (Ti) catalyst used for the initial experiments, as well as the industry standard antimony(III) oxide (Sb). Reaction times of 5 and 22 h (in addition to the 2 h initial reaction at 150 °C described in the experimental section) were evaluated, and all reactions of recovered TPA were benchmarked against commercial pure TPA reacted under the same conditions.

As was observed in the primary screening, NMR revealed that the reaction mixtures contained BHET (and possibly mono-2-hydroxyethyl terephthalate (MHET)) along with (c)OET, in addition to unreacted TPA and EG (Figure S24). Moreover, some material was insoluble in DMSO, rather than in a mixture of TFA and CDCl_3_, indicating PET formation.

**Figure 3:**
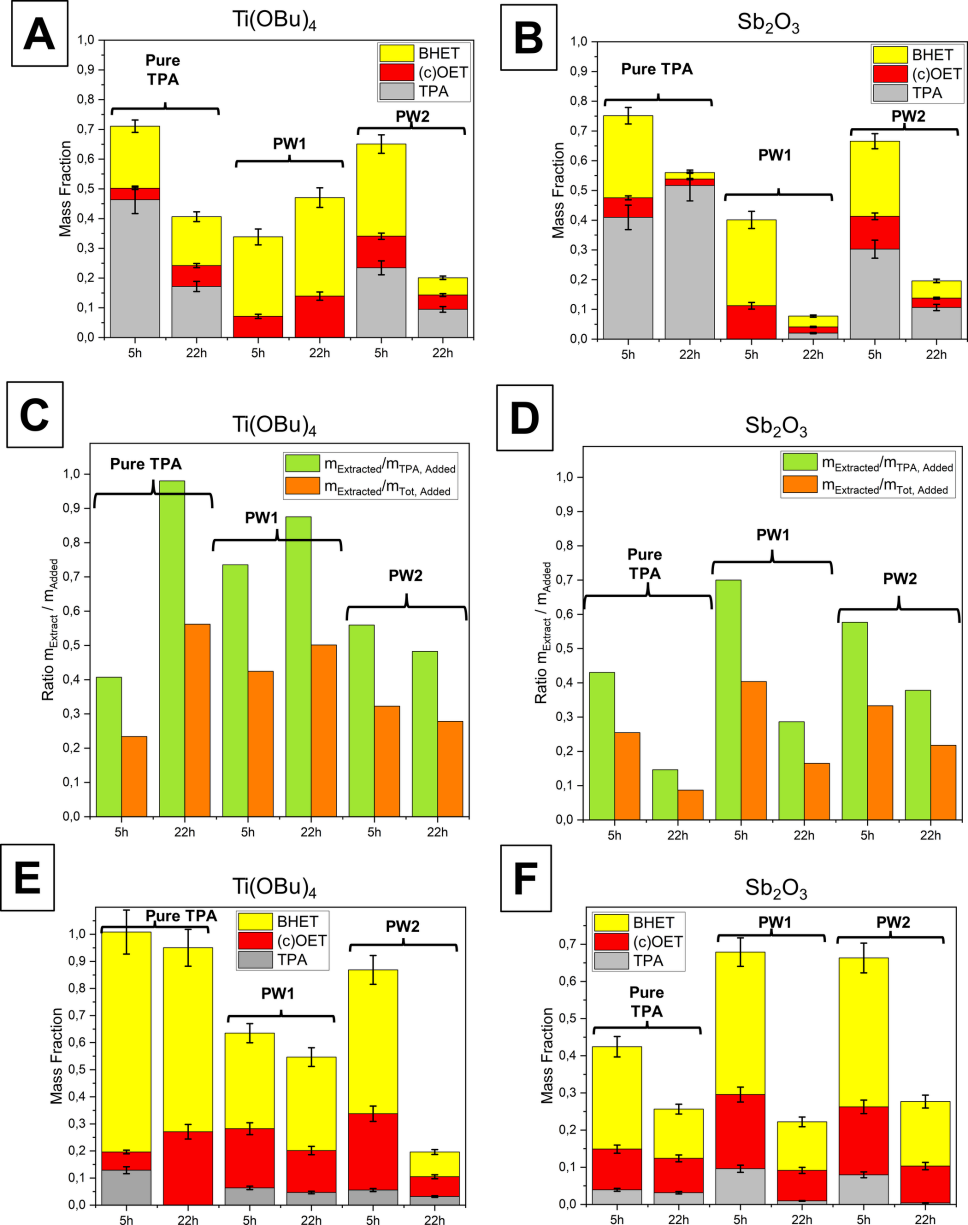
Effect of catalyst and reaction time on the content of Bis(2-hydroxyethyl terephthalate), BHET, (cyclo)(oligoethylene terephthalate), c(OET), and terephthalic acid, TPA, after reaction of commercial TPA and recovered fractions PW1 and PW2 as determined by Q-NMR. A) Mass composition of DMSO-soluble individual components after reaction using the Ti(OBu)_4_ catalyst. B) Mass composition of DMSO-soluble individual components after the reaction using the Sb_2_O_3_ catalyst. C) Amount of material extracted in 150 °C warm anisole relative to added amounts of TPA and the total amount for the reaction using the Ti(OBu)_4_ catalyst. D) Amount of material extracted in 150 °C warm anisole relative to added amounts of TPA and the total amount for the reaction using the Sb_2_O_3_ catalyst. E) Mass composition of individual components after extraction with anisole at 150 °C using the Ti(OBu)_4_ catalyst. F) Mass composition of individual components after extraction with anisole at 150 °C using the Sb_2_O_3_ catalyst. Error bars in A, B and E, F correspond to 10% of the relative fraction based on an estimated uncertainty of ^1^H-NMR integrals.

The amounts of each component were assessed by using quantitative NMR. Figure 3A shows that when pure TPA was used in the presence of the Ti catalyst, the amount of residual TPA was significant after 5 h, with a combined BHET and soluble oligomer content of only around 20%. This was lower than the initial results (Figure S23). However, the above-mentioned experiments were performed on a 0.5 g TPA scale in 4 mL vials fitted with a condenser with a significantly larger headspace compared to the low-volume initial screening conducted in NMR tubes. This may lead to a larger fraction of the volatile EG being in a gaseous state or on reactor walls and therefore not participating in the reaction.

##### Reaction Time Effect, Pure TPA

For pure TPA (see Figure 3A and B), longer heating times reduced the amount of unreacted TPA, and the amount of DMSO-soluble material decreased, directly indicating increased PET formation (PET is insoluble in DMSO). The two catalysts showed little difference with pure TPA after 5 h according to QNMR (compare Figure 3A and B). Nevertheless, after 22 h of heating, the Sb-catalyzed reaction resulted in less soluble material, especially BHET and (c)OET, indicating that the antimony catalyst is a superior polycondensation catalyst. The residual soluble material mostly consists of TPA, illustrating that the catalyst is relatively poor for the initial esterification between EG and TPA.

##### Crude TPA

For the crude samples PW1 and PW2 (Figure 3A and B), longer heating times reduced the amount of unreacted TPA and, except for PW1 using the Ti catalyst, the amount of DMSO-soluble material decreased, demonstrating increased PET formation. For PW1 in the presence of the Ti catalyst, an increase in soluble (c)OET was observed with longer heating times, which probably reflects the excess EG suppressing polymer formation as expected.

For PW1, the total amount of soluble material was less than 10% after 22 h when using Sb but close to 50% when using Ti. This follows the trend observed for pure TPA, although the amount of residual TPA was significantly lower for the crude material due to a larger relative amount of EG. On the other hand, the reactions based on PW2-derived TPA with both catalysts were similar in terms of initial esterification and subsequent transesterification efficiency. However, they differed significantly from the reaction with pure TPA, where essentially all polymerizable material (BHET and (c)OET) had disappeared after 22 h when using the Sb catalyst, suggesting that the present impurities (a combination of acid in a salt form and minor amounts of at least one unidentified colored product, which is likely to be oxidized aromats^41^) affected the polymerization. This difference was less distinct for the samples using the Ti catalyst, which is consistent with this catalyst being a less efficient polycondensation catalyst.

Analysis of the 5 h results by size exclusion chromatography (Figure S25A) revealed that the titanium-based catalyst gives shorter chains than the antimony-based catalyst for both the pure TPA and for PW2, again emphasizing that the latter is a more efficient polymerization catalyst. In the case of PW1, the molecular weight averages are comparable for the two catalysts suggesting deactivation of the (polycondensation) activity of the antimony catalyst.

### Extraction of BHET and (c)OETs

To remove any residual salts and excess TPA, the reaction mixtures were extracted with anisole at 150 °C. At this temperature, anisole is a good solvent for BHET and (c)OET as well as for EG and the catalyst but remains a poor solvent for TPA and salts and longer polyesters. This insoluble fraction could easily be filtered off. In addition, anisole is considered benign,^42^ and due to its relatively high vapor pressure, it can, in principle, be recovered and reused. Figure 3C and D shows the ratios of extracted material relative to the amount of TPA and total added material.

#### Extraction Efficiency

The discrepancy between the apparent mass fractions in Figure 3A and B and the extracted amounts in Figure 3C and D probably reflects differences in solubility; DMSO solubilizes TPA, BHET, and short (c)OETs but not PET. On the other hand, warm anisole may be capable of dissolving (shorter) PET, thus allowing a significant amount of DMSO-insoluble PET to be extracted. The Sb-catalyzed reactions resulted in a total amount of extracted material decreasing with increasing reaction time, supporting that this catalyst more efficiently promotes polycondensation and, thereby, the formation of polymer chains too long to be soluble in warm anisole.

Figure 3E and F shows the composition of the extracted material measured by Q-NMR in DMSO. In all samples, the TPA content was significantly reduced compared to the material before extraction. It should also be noted that EG was observed in these samples (Figure S26) as would be expected.

#### Extract Composition, Ti Catalyst

For the Ti catalyst with pure TPA and PW2, the main components after 5 h were BHET and oligomers, whereas PW1 contained a larger amount of the insoluble material. After 22 h, the extracted amount still mostly consisted of BHET and (c)OET from the pure TPA, although the overall extracted amount was lower (Figure 3C and D), indicating significant formation of longer, less soluble chains. This was further emphasized by a larger relative content of (c)OET. For PW1, the composition was similar after 5 and 22 h, suggesting catalyst deactivation, whereas PW2 showed only a small amount of soluble material after 22 h, indicating significant formation of chains that are insoluble in DMSO but can be extracted in warm anisole.

#### Extract Composition, Sb Catalyst

The use of the Sb catalyst led to a decrease in extracted material after 22 h compared to 5 h for all samples, supporting the polycondensation efficiency of this catalyst. As expected, the efficiency appears to be higher for pure TPA.

A potential issue with using this method for BHET formation is that BHET is somewhat volatile and, thus, that there may be significant losses during the removal of anisole and subsequent drying. In principle, some loss of BHET may be caused through coevaporation with anisole. However, the large relative amount of extracted BHET from the pure TPA reaction indicates that manipulation-related losses are relatively low, in agreement with literature reports.^43^ In addition, during the cooling of the extracted and filtered solution, crystallization was observed, implying that filtration may suffice in isolating most of the extracted material, and this method is preferential due to less energy consumption.

To summarize, the anisole extraction is efficient for removal of salts when using the Ti catalyst, since this catalyst primarily forms BHET and oligomers that are soluble in warm anisole. However, the extraction procedure is less useful for the Sb catalyst because this is a more efficient polycondensation catalyst, forming longer, less soluble chains.

### Repolymerization

To test the use of the mixtures in the preparation of PET, a two-step procedure was applied. First, the crude mixtures after a 5 h reaction and extraction with anisole (assumed to still contain a catalyst since this is expected to be extracted into the anisole fraction) were heated to 280 °C for 15 min under nitrogen. These conditions have been reported to be efficient for the rapid ring-opening polymerization (ROP) of cyclic oligoethylene terephthalates^40^ and will also remove any residual ethylene glycol. In addition, the polycondensation of BHET is expected to occur. Subsequently, the crude mixtures from the ROP were placed at 5 mbar at 200 °C for 12 h to explore the possibility of solid-state polymerization through polycondensation.^44^ The resulting product was analyzed by size exclusion chromatography without further purification steps (Figure S25), allowing assessment of molecular weight averages. Both the titanium- or the antimony-based catalysts present in the material were seen to polymerize the extracts based on the results from the pure TPA (see above). For the crude TPAs, the antimony-based catalyst was again found to be superior, resulting in larger molecular weights. The recovered samples resulted in a lower apparent degree of polymerization for both catalysts (see Figure S27), again with the antimony-based catalyst generally leading to longer chains. It should be emphasized that these are pilot-scale experiments mainly serving to demonstrate the possibility of repolymerization and that no optimization studies were undertaken. In light of the results outlined above, it appears that the presence of impurities suppresses or retards the activity of the catalyst but that it does not completely deactivate it. Therefore, a setup where the catalyst amount reflects a measured amount of impurities could be expected to lead to larger polymers.

## Conclusion

Enzyme catalyzed recovery of TPA from PET rich plastic waste samples and from pure polyesters using enzymes was demonstrated. Thereby, restriction well known for the commercial HiC regarding crystalline materials could be overcome with novel enzymes recently developed by several groups. The different PET and PBAT hydrolysates were acidified to successfully recover TPA from realistic plastic wastes, which resulted in considerable amounts of recovered monomer (8-11 g per 40 g of initial polymer waste) with high purity (>80%), whereas TPA recovered from pure PET samples was >90% pure.

The recovered TPA could be reacted directly with ethylene glycol to prepare PET precursors bis(2-hydroxyethyl) terephthalate and oligo(ethylene terephthalate). A simple extraction method allowed isolation of these compounds in good purities, and an increase in molecular weight was demonstrated using standard methods for PET preparation, demonstrating the possibility of using the recovered TPA for preparation of PET with little further purification. Altogether these findings suggest new perspectives for the recovery of building blocks from wastes containing different polymers, contaminants, and pigments.

## Supporting Information Available

Materials and methods sections, calibration curves and additional characterizations of both hydrolyzed polymers and recovered monomers (HPLC, DSC, FT-IR, NMR spectra), size exclusion chromatography results of the resynthesis reaction (PDF)

## References

[ref1] European Commission. European Green Deal: Putting an end to wasteful packaging, boosting reuse and recycling. Published 2022. Accessed November 16, 2023. https://ec.europa.eu/commission/presscorner/detail/en/ip_22_7155.

[ref2] European Commission. The European Green Deal. Striving to be the first climate-neutral continent. Published 2023. Accessed November 17, 2023. https://commission.europa.eu/strategy-and-policy/priorities-2019-2024/european-green-deal_en.

[ref3] Kawai F.; Kawabata T.; Oda M. Current knowledge on enzymatic PET degradation and its possible application to waste stream management and other fields. Appl. Microbiol Biotechnol. 2019, 103 (11), 4253–4268. 10.1007/s00253-019-09717-y. 30957199 PMC6505623

[ref4] Witt U.; Müller RJ; Deckwer WD. Biodegradation behavior and material properties of aliphatic/aromatic polyesters of commercial importance. J. Environ. Polym. Degrad. 1997, 5 (2), 81–89. 10.1007/BF02763591.

[ref5] Du Toit A. Plastic communities. Nat. Rev. Microbiol. 2022, 20 (10), 575 10.1038/s41579-022-00790-1. 35970868

[ref6] Müller RJ; Schrader H.; Profe J.; Dresler K.; Deckwer WD. Enzymatic degradation of poly(ethylene terephthalate): Rapid hydrolyse using a hydrolase from T. fusca. Macromol. Rapid Commun. 2005, 26 (17), 1400–1405. 10.1002/marc.200500410.

[ref7] Ribitsch D.; Yebra AO; Zitzenbacher S.; et al. Fusion of binding domains to Thermobifida cellulosilytica cutinase to tune sorption characteristics and enhancing PET hydrolysis. Biomacromolecules 2013, 14 (6), 1769–1776. 10.1021/bm400140u. 23718548

[ref8] Tournier V.; Topham CM; Gilles A.; et al. An engineered PET depolymerase to break down and recycle plastic bottles. Nature 2020, 580 (7802), 216–219. 10.1038/s41586-020-2149-4. 32269349

[ref9] Sonnendecker C.; Oeser J.; Richter PK; et al. Low Carbon Footprint Recycling of Post-Consumer PET Plastic with a Metagenomic Polyester Hydrolase. ChemSusChem. 2022, 15 (9), e202101062 10.1002/cssc.202101062. 34129279 PMC9303343

[ref10] Urbanek AK; Mirończuk AM; García-Martín A.; Saborido A.; de la Mata I.; Arroyo M. Biochemical properties and biotechnological applications of microbial enzymes involved in the degradation of polyester-type plastics. Biochim Biophys Acta - Proteins Proteomics 2020, 1868 (2), 140315 10.1016/j.bbapap.2019.140315. 31740410

[ref11] Plastics Europe. Plastics-the fast Facts 2023. Published 2023. Accessed November 13, 2023. https://plasticseurope.org/knowledge-hub/plastics-the-fast-facts-2023/.

[ref12] Sousa AF; Patrício R.; Terzopoulou Z.; et al. Recommendations for replacing PET on packaging, fiber, and film materials with biobased counterparts. Green Chem. 2021, 23 (22), 8795–8820. 10.1039/D1GC02082J.

[ref13] Abid U.; Sun G.; Soong YHV; et al. Evaluation of enzymatic depolymerization of PET, PTT, and PBT polyesters. Biochem Eng. J. 2023, 199 (July), 109074 10.1016/j.bej.2023.109074.

[ref14] Jian J.; Xiangbin Z.; Xianbo H. An overview on synthesis, properties and applications of poly(butylene-adipate-co-terephthalate)–PBAT. Adv. Ind. Eng. Polym. Res. 2020, 3 (1), 19–26. 10.1016/j.aiepr.2020.01.001.

[ref15] Lee HL; Chiu CW; Lee T. Engineering terephthalic acid product from recycling of PET bottles waste for downstream operations. Chem. Eng. J. Adv. 2021, 5 (October 2020), 100079 10.1016/j.ceja.2020.100079.

[ref16] Collias DI; Harris AM; Nagpal V.; Cottrell IW; Schultheis MW. Biobased terephthalic acid technologies: A literature review. Ind. Biotechnol. 2014, 10 (2), 91–105. 10.1089/ind.2014.0002.

[ref17] He Y.; Luo Y.; Yang M.; et al. Selective catalytic synthesis of bio-based terephthalic acid from lignocellulose biomass. Appl. Catal. A Gen. 2022, 630 (November 2021), 118440 10.1016/j.apcata.2021.118440.

[ref18] Ogunjobi JK; Farmer TJ; McElroy CR; et al. Synthesis of Biobased Diethyl Terephthalate via Diels-Alder Addition of Ethylene to 2,5-Furandicarboxylic Acid Diethyl Ester: An Alternative Route to 100% Biobased Poly(ethylene terephthalate). ACS Sustain Chem. Eng. 2019, 7 (9), 8183–8194. 10.1021/acssuschemeng.8b06196.

[ref19] Rostovtseva V.; Faykov I.; Pulyalina A. A Review of Recent Developments of Pervaporation Membranes for Ethylene Glycol Purification. Membranes (Basel) 2022, 12 (3), 312 10.3390/membranes12030312. 35323787 PMC8956067

[ref20] Ismail M.; Abouhmad A.; Warlin N.; et al. Closing the loop for poly(butylene-adipate-co-terephthalate) recycling: depolymerization, monomers separation, and upcycling. Green Chem. 2024, 26 (7), 3863–3873. 10.1039/D3GC04728H.

[ref21] Brackmann R.; de Oliveira Veloso C.; de Castro AM; Langone MAP. Enzymatic post-consumer poly(ethylene terephthalate) (PET) depolymerization using commercial enzymes. 3 Biotechnol. 2023, 13 (5), 135 10.1007/s13205-023-03555-6. PMC10130296 37124991

[ref22] Quartinello F.; Vajnhandl S.; Volmajer Valh J.; et al. Synergistic chemo-enzymatic hydrolysis of poly(ethylene terephthalate) from textile waste. Microb Biotechnol. 2017, 10 (6), 1376–1383. 10.1111/1751-7915.12734. 28574165 PMC5658601

[ref23] Deshoulles Q.; Gall M Le; Benali S.; et al. Hydrolytic degradation of biodegradable poly(butylene adipate-co-terephthalate) (PBAT) - Towards an understanding of microplastics fragmentation. Polym. Degrad Stab. 2022, 205 (June), 110122 10.1016/j.polymdegradstab.2022.110122.

[ref24] Pang W.; Li B.; Wu Y.; et al. Upgraded recycling of biodegradable PBAT plastic: Efficient hydrolysis and electrocatalytic conversion. Chem. Eng. J. 2024, 486 (March), 150342 10.1016/j.cej.2024.150342.

[ref25] Weinberger S.; Canadell J.; Quartinello F.; et al. Enzymatic degradation of poly(Ethylene 2,5-furanoate) powders and amorphous films. Catalysts 2017, 7 (11), 318 10.3390/catal7110318.

[ref26] Ribitsch D.; Yebra AO; Zitzenbacher S.; et al. Fusion of binding domains to Thermobifida cellulosilytica cutinase to tune sorption characteristics and enhancing PET hydrolysis. Biomacromolecules 2013, 14 (6), 1769–1776. 10.1021/bm400140u. 23718548

[ref27] Gritsch SM; Mihalyi S.; Bartl A.; et al. Closing the cycle: Enzymatic recovery of high purity glucose and polyester from textile blends. Resour Conserv Recycl. 2023, 188 (July 2022), 106701 10.1016/j.resconrec.2022.106701.

[ref28] Novozymes. Esterase, Liquid StickAway®. https://www.novozymes.com/en/products/pulp-paper/stickaway.

[ref29] Wang Q.; Liu S.; Yang G.; Chen J.; Ji X.; Ni Y. Recycling cellulase towards industrial application of enzyme treatment on hardwood kraft-based dissolving pulp. Bioresour Technol. 2016, 212, 160–163. 10.1016/j.biortech.2016.04.048. 27099940

[ref30] Lindedam J.; Haven MØ; Chylenski P.; Jørgensen H.; Felby C. Recycling cellulases for cellulosic ethanol production at industrial relevant conditions: Potential and temperature dependency at high solid processes. Bioresour Technol. 2013, 148, 180–188. 10.1016/j.biortech.2013.08.130. 24045205

[ref31] Donelli I.; Taddei P.; Smet PF; Poelman D.; Nierstrasz VA; Freddi G. Enzymatic surface modification and functionalization of PET: A water contact angle, FTIR, and fluorescence spectroscopy study. Biotechnol Bioeng. 2009, 103 (5), 845–856. 10.1002/bit.22316. 19365872

[ref32] Siracusa C.; Quartinello F.; Soccio M.; et al. On the Selective Enzymatic Recycling of Poly(pentamethylene 2,5-furanoate)/Poly(lactic acid) Blends and Multiblock Copolymers. ACS Sustain Chem. Eng. 2023, 11 (26), 9751–9760. 10.1021/acssuschemeng.3c01796. 37425282 PMC10324456

[ref33] Chen Y.; Zou H.; Liang M.; Cao Y. Melting and crystallization behavior of partially miscible high density polyethylene/ethylene vinyl acetate copolymer (HDPE/EVA) blends. Thermochim Acta. 2014, 586, 1–8. 10.1016/j.tca.2014.04.007.

[ref34] De Rosa C.; Auriemma F.; Vinti V.; Galimberti M. Equilibrium melting temperature of syndiotactic polypropylene. Macromolecules 1998, 31 (18), 6206–6210. 10.1021/ma9805248.

[ref35] Prasad A. A quantitative analysis of low density polyethylene and linear low density polyethylene blends by differential scanning calorimetery and fourier transform infrared spectroscopy methods. Polym. Eng. Sci. 1998, 38 (10), 1716–1728. 10.1002/pen.10342.

[ref36] Panasyuk GP; Azarova LA; Khaddaj M.; et al. Preparation and properties of sodium, potassium, magnesium, calcium, and aluminum terephthalates. Inorg. Mater. 2003, 39 (12), 1292–1297. 10.1023/B:INMA.0000008916.84994.e1.

[ref37] Sabu T.; Visakh P. M. Handbook of Engineering and Speciality Thermoplastics, Vol. 3: Polyethers; 2011.

[ref38] Goodman I.; Nesbitt BF. The structures and reversible polymerization of cyclic oligomers from poly(ethylene terephthalate). Polymer (Guildf) 1960, 1 (C), 384–396. 10.1016/0032-3861(60)90048-3.

[ref39] Peebles LH; Ablett CT.; et al. Isolation and Identification of the. J. Polym. Sci. 1969, 7, 479–496. 10.1002/pol.1969.150070207.

[ref40] Burch RR; Lustig SR; Spinu M. Synthesis of cyclic oligoesters and their rapid polymerization to high molecular weight. Macromolecules 2000, 33 (14), 5053–5064. 10.1021/ma000278b.

[ref41] Ladasiu Ciolacu C. F.; Roy Choudhury N.; Dutta NK. Colour formation in poly(ethylene terephthalate) during melt processing. Polym. Degrad Stab. 2006, 91 (4), 875–885. 10.1016/j.polymdegradstab.2005.06.021.

[ref42] Byrne F. P.; Jin S.; Paggiola G.; Petchey T. H. M.; Clark J. H.; Farmer T. J.; Hunt A. J.; McElroy C. R.; Sherwood JR. Tools and techniques for solvent selection: green solvent selection guides. Sustain Chem. Process 2016, 4 (1), 7 10.1186/s40508-016-0051-z.

[ref43] Chen Z.; Sun H.; Kong W.; Chen L.; Zuo W. Closed-loop utilization of polyester in the textile industry. Green Chem. 2023, 25, 4429–4437. 10.1039/D3GC00407D.

[ref44] Ma Y.; Agarwal US; Sikkema DJ; Lemstra PJ. Solid-state polymerization of PET: Influence of nitrogen sweep and high vacuum. Polymer (Guildf) 2003, 44 (15), 4085–4096. 10.1016/S0032-3861(03)00408-7.

