# Supplementary material for: There and Back Again: Recovery of Terephthalic Acid from Enzymatically Hydrolyzed Polyesters for Resynthesis
Source: ACS Sustain Resour Manag. 2025 Jan 23;2(2):334–42. doi: 10.1021/acssusresmgt.4c00430 (PMC11874462; doi:10.1021/acssusresmgt.4c00430)
Supplement: Supplementary file 1 — rm4c00430_si_001.pdf [file rm4c00430_si_001.pdf]

Electronic Supplementary Materials

There and back again: recovery of terephthalic acid from enzymatically hydrolysed polyesters for resynthesis

Chiara Siracusa[1,2], Virginia Celestre[3], Felice Quartinello[1,2], Giacomo Damonte[4], Jeppe Madsen[3], Georg M. Guebitz[1,2], Anders Egede Daugaard[3,*], Alessandro Pellis[1,2,4,*]

[1] acib GmbH, Konrad-Lorenz-Strasse 20, 3430 Tulln an der Donau, Austria.

[2] Institute of Environmental Biotechnology, University of Natural Resources and Life Sciences Vienna Konrad-Lorenz-Strasse 20, 3430 Tulln an der Donau, Austria.

[3] Danish Polymer Centre, Department of Chemical and Biochemical Engineering, Technical University of Denmark, Lyngby, Denmark

[4] Department of Chemistry and Industrial Chemistry, Università degli Studi di Genova, Via Dodecaneso 31, 16146 Genova, Italy

* Correspondence to: Prof. Anders E. Daugaard, adt@kt.dtu.dk and Prof. Alessandro Pellis, alessandro.pellis@unige.it

Materials

Chemicals and substrates

Synthetic polyester films poly(butylene adipate-*co*-butylene terephthalate) (PBAT) abbreviated here as PBAT1, amorphous poly(ethylene terephthalate) abbreviated as PET1 and PET2 were provided by BioMi (Matulji, Croatia). Additional samples, PET 3, and PBAT2 were purchased respectively from GoodFellow and BASF (BASF SE, Ludwigshafen, Germany). Mixed waste samples containing PET were supplied by AIMPLAS (Mixed plastic waste bottles Origin Spain (size 1 mm) with a PET content of 98.6%, abbreviated here as PW1, and mixed plastic waste trays (sized to 1 mm) with predominantly containing PET, hereby PW2).

Di-potassium hydrogen phosphate (K_2HPO_4) for buffer preparation was provided by Roth (Germany). Terephthalic acid (Pure TPA, 98%) was provided by Merck. Other chemicals and pure monomers for instrument calibration and purity comparison were purchased by Sigma-Aldrich (adipic acid (AA), 1,4-butanediol (BDO), ethylene glycol (EG) (anhydrous, 99.8%)). The commercially available enzyme *Humicola insolens* cutinase (hereby abbreviated as HiC) is a product from Novozymes.

Titanium (IV) butoxide (97%), antimony trioxide (99.999%), maleic acid (MA, >99.0 %) and trifluoroacetic acid (TFA, ≥99.0%) were obtained from Sigma-Aldrich (Merck) and used as received. Dichloromethane (Ph. Eur. BP, NF) and dimethyl sulfoxide (DMSO, ≥99.7%) was obtained from Sigma-Aldrich (Merck). 1,1,1,3,3,3-Hexafluoropropan-2-ol (HFIP, >99.0%) N-Methylmaleimide (>98.0%) was obtained from TCI. Deuterated Chloroform (99.8%) was purchased from VWR.

Methods

Enzyme Characterization: Bradford Assay for Protein Content and Activity Assay

Bradford spectrophotometric protein assay was applied to determine the enzyme concentration in the stock commercial solution before use. Water was used as a blank, and Bovine Serum Albumine (BSA) as a reference. The activity of the enzyme was assessed through esterase activity assay with *para*-nitrophenylbutyrate (*p*-NPB) as model substrate. Both assays were performed as previously described[1].

Sample Preparation and Enzymatic Hydrolysis

Different size polymer films (strips of 0.5 cm × 3.0 cm strips or 10 cm × 3.0 cm) were cut, washed in three subsequent steps as suggested in Bertolini FA et al. (2021)[2] to remove possible impurities: Triton X-100 (2 g/L), Na_2CO_3 and a final rinsing with ultrapure water. A first characterization step was carried out to determine the initial weight and other surface features prior to the treatment. Details about the initial weight of each differently sized films are listed in Table S1.

Table S1. Initial weight of different sized films.

Sample	Size (cm x cm)	Average Weight (mg)
**PET1**	0.5 × 3.0	28.50±0.26
	10 × 3.0	454.39±1.39
**PET2**	0.5 × 3.0	2.47±0.49
	10 × 3.0	36.65±3.21
**PET3**	0.5 × 3.0	44.10±0.61
	10 × 3.0	890.12±3.54
**PBAT1**	0.5 × 3.0	8.89±0.43
	10 × 3.0	143.33±4.52
**PBAT2**	0.5 × 3.0	12.63±0.38
	10 × 3.0	245.45±5.25

The incubation was performed in Potassium Phosphate 1 M (K_2HPO_4/KH_2PO_4) buffer adjusted to pH = 8 according to previously established conditions[1], where HiC was added to a final concentration of 5 µM if not differently specified[3]. Smaller size films were incubated in 2 mL Eppendorf tubes (65° C and 150 rpm agitation in Orbital Shaker) while the 10 cm × 3.0 cm strips were placed in 50 mL buffer volume in glass bottles and incubated at the same conditions. All reactions were monitored through time point collection after 6 h, 24 h, 48 h and 72 h. At the same time blanks for each film type were incubated with buffer only. Each experiment was performed in triplicate. The mixed waste was instead incubated as fragments (1 mm average size). A first experiment was carried out with 2 g of material in 100 mL incubation solution. Afterwards, the reaction was upscaled to 40 g in 2 L bottle (20 g substrate per 1 L of solution). Additional experiments of PET hydrolysis were performed in parallel for enzyme quantity optimization (ranging HiC concentration from 5 µM to 15 µM). The reactions were stopped after the incubation through samples storage at 4 °C and methanol precipitation. Hydrolysates were acidified (20 µL of 6 N HCl per 1 mL solution), centrifuged (14000 rpm, 4°C, 15 minutes) and filtered (0.2 µm polyamide filters). Samples were characterized in weight, surface features and crystallinity before and after the treatment.

Enzymatic hydrolysis reaction assessment

Weight loss and surface characterization of partially degraded samples (FT-IR) and DSC

Post-incubation residual films or fragments were washed and dried according to the same procedures described above. First, their weight loss was measured, and a gravimetric determination was carried out. Fourier Transform Infrared Spectroscopy (FT-IR, PerkinElmer Spectrum 100 Spectrometer in ATR mode) was then used to characterize the surface functional groups. Spectra were recorded from 650 cm^{-1} to 4000 cm^{-1}, at a resolution of 2 cm^{-1} for 40 scans. Chemical groups assignment was performed by comparing the sample peaks to the blank peaks and a reference table. The films after hydrolysis were also analysed through Differential Scanning Calorimetry (DSC) for overall crystallinity variation assessment.

Quantification of soluble released products *via* HPLC

High Performance Liquid Chromatography (HPLC) (Agilent Technologies, 1260 Infinity equipped with a reversed phase column C18 (YMC 30, 250 × 4.6 mm ID, S-5 µm) was used to measure monomers concentration in each time point hydrolysates. TPA was identified by UV detection at 245 nm. The flow rate of methanol and 0.1% formic acid was kept constant at 0.85 mL min^{-1}. Each sample was prepared in triplicate, by diluting accordingly in ice cold methanol. 30 µL 6 N HCl were added to the samples, centrifuged for 15 minutes (14000 rpm, 4° C) before being filtered (0.2 µm polyamide filter) into HPLC vials. Aliphatic compounds (adipic acid, 1,4 butanediol, ethylene glycol) were instead quantified using a refractive index detector (Transgenomic IC SEP-ION-300). The procedure for HPLC preparation was based on Carrez precipitation[4], to clear the hydrolysate from proteins. The solutions added sequentially are prepared from potassium hexacyanoferrate (II) trihydrate and zinc sulphate heptahydrate. Centrifugation for 30 minutes (at 14000 rpm, 4° C) was carried out, before filtration into HPLC vials. The mobile phase for the analysis was 0.01 N H_2SO_4 (flow rate 0.325 mL min^{-1}). For both aliphatic and aromatic monomers, calibrations prepared in advance were used to correlate the signal intensity to the concentration expressed in mM. Calibration curves and the associated tables are reported in Figure S1 and Table S2.

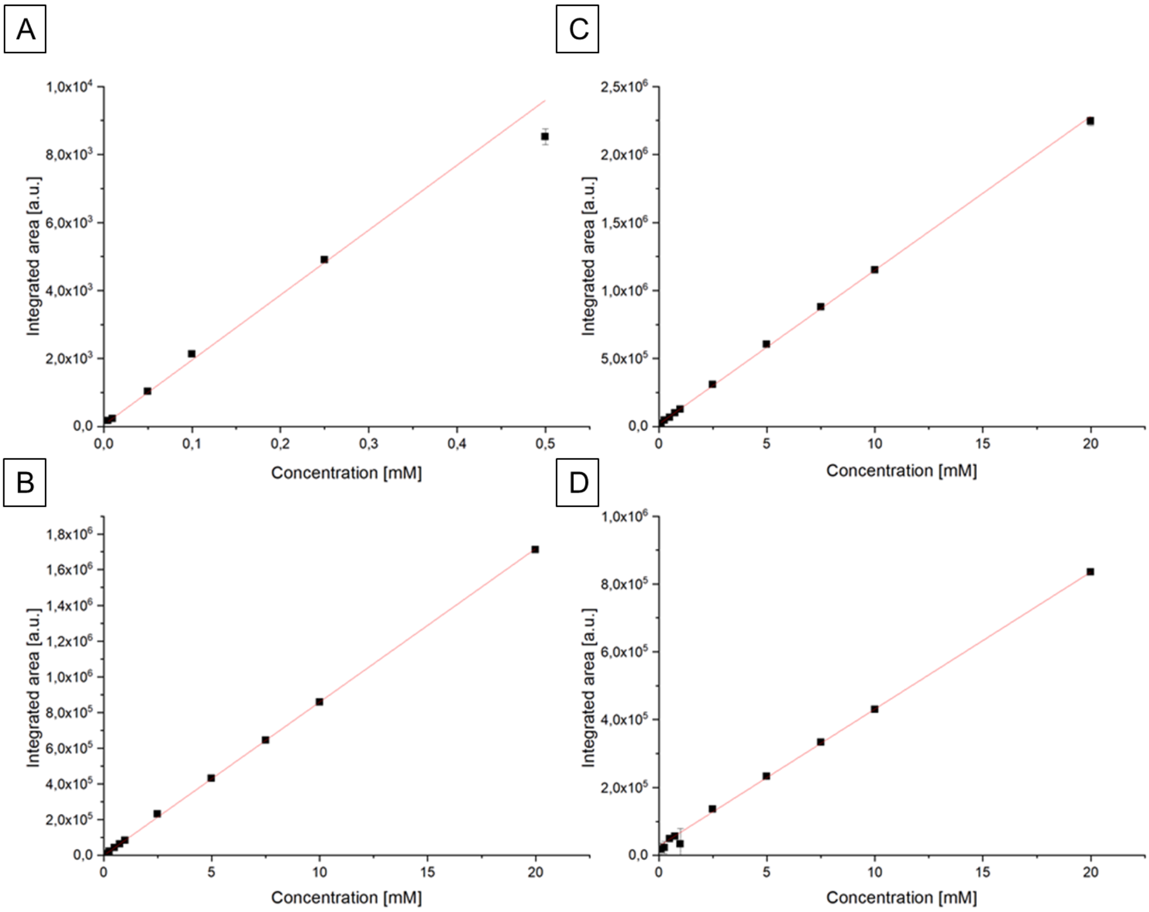

Figure S1. Calibration curves for A: terephthalic acid (TPA); B: 1,4-butanediol (BDO); C: adipic acid (AA); D: ethylene glycol (EG)

Table S2. Concentration (expressed in mM) associated with the respective area average measured *via* HPLC for the determination of standard curves for each monomer

**Adipic acid**	**Eq. y = 111906x + 21629**	
**Concentration [mM]**	**Avg Area**	**St.dev. Area**
**20**	2243143.40	31588.00
**10**	1149360.85	1092.61
**7.5**	879213.25	6256.03
**5**	602936.85	9776.55
**2.5**	309100.60	3177.36
**1**	125445.65	11822.60
**0.75**	99731.35	6352.42
**0.5**	67033.35	8736.81
**0.25**	46175.40	1093.23
**0.1**	20887.90	7910.50

**1.4-butanediol**	**Eq. y = 85490x + 2631.5**	
**Concentration [mM]**	**Avg Area**	**St.dev. Area**
**20**	1710414.50	2059.66
**10**	858049.50	2918.23
**7.5**	643599.50	468.95
**5**	431520.10	476.17

**2.5**	231538.05	11074.92
**1**	84439.05	417.26
**0.75**	63718.75	572.83
**0.5**	42696.95	662.63
**0.25**	21155.80	2.83
**0.1**	8520.05	218.43

**Ethylen glycol**	**Eq. y = 41075x + 18818**	
**Concentration [mM]**	**Avg Area**	**St.dev. Area**
**20**	834970.90	233.63
**10**	430097.85	1881.12
**7.5**	332949.40	603.44
**5**	232192.40	1719.12
**2.5**	135778.65	7028.15
**1**	33747.50	45916.12
**0.75**	55339.20	591.57
**0.5**	48268.00	636.82
**0.25**	22904.05	17516.80
**0.1**	17103.15	17848.58

**Terephthalic acid**	**Eq. y = 17046x + 229.92**	
**Concentration [mM]**	**Avg Area**	**St.dev. Area**
**0.5**	8523.00	233.00
**0.25**	4895.83	70.09
**0.1**	2125.17	58.53
**0.05**	1028.27	19.81
**0.01**	233.10	4.81
**0.005**	171.57	13.39

Monomer recovery and Purity evaluation

The precipitation was induced gradually, by a step-by-step acidification procedure. 6 N HCl was added in 1 mL aliquots, until a final pH of 2 was reached. Between each addition, the solution was stirred and the pH was monitored. The decrease of TPA solubility was followed by the formation of white precipitate. After each addition, a sample was also withdrawn and centrifuged to measure the residual soluble TPA via HPLC.

After mixing the solution, a centrifugation step (20 minutes, 3200 rpm, 4° C) followed, until a precipitated fraction was separated from the clear supernatant. The pellet was then resuspended in ultrapure water, adjusted to pH = 2 and kept on a stirring plate (heated to 80° C) overnight. An additional precipitation step was carried out as described above and the pellet was subsequently freeze-dried and analysed through

Fourier-Transform Infrared Spectroscopy (FT-IR), Proton Nuclear Magnetic Resonance spectroscopy (^{1}H-NMR) and Thermogravimetric Analysis (TGA). All the experiments were performed in triplicates and the supernatants were checked for residual terephthalic acid.

^{1}H-NMR spectroscopy was performed using a JEOL ECZ400R/S3 at a frequency of 400 MHz, 8 scans, using DMSO-d_6 as the solvent at room temperature. ~10mg of material were dissolved in 0.7 mL of the NMR solvent.

FT-IR spectra were collected through a PerkinElmer ATR-FT-IR spectrometer Spectrum One. Each measurement was performed in a 650–4000 cm^{-1} wavelength range (2 cm^{-1} resolution, 40 scans). The spectra were then acquired and processed by the software PerkinElmer data manager (Spectrum).

Thermogravimetric analysis (TGA) was performed using a Mettler Toledo “TGA/DSC1 STARe System®” instrument. In detail, 10 mg of sample were loaded into a 100 µL alumina crucible and analyzed in the range from 30 °C to 800 °C, using a heating rate of +10 °C min^{-1} and under a nitrogen flow of 80 mL min^{-1}. Temperatures of initial degradation (T_{onset}) and of the maximum degradation rate (T_{max}) were determined using Mettler Toledo STARe® software.

Differential Scanning Calorimetry (DSC) was performed using a Mettler Toledo “DSC1 STARe System®” instrument. Specifically, 5 mg of sample were inserted into a 40 µL aluminium pan with perforated lid and scanned in the temperature range from 30 °C to 300 °C, using a heating rate of +10 °C min^{-1} and a nitrogen flow of 20 mL min^{-1}. The glass transition temperature (T_g) was determined using STARe® software and the crystallinity degree (χ_c) of the samples was calculated using Equation 1, using cold crystallization enthalpy (ΔH_{cc}) and melting enthalpy (ΔH_m) values measured from the first heating scan.

$$\chi_c = \frac{\Delta H_m - \Delta H_{cc}}{\Delta H_m^0} \cdot 100 \quad \text{(Equation 1)}$$

Where ΔH_m is the measured melting enthalpy, and ΔH_m^0 is the melting enthalpy of a 100% crystalline PET, considered as 140/ J/g[5].

Quantitative NMR (Q-NMR) samples were prepared by weighing 10-50 mg of TPA for reaction into an NMR tube. Internal standards of maleic acid or N-methyl maleimide were used to assess respectively TPA purity and content in reaction mixtures. The

method was validated using commercial TPA, with a stated purity of 98 %. All ^{1}H analyses for Q-NMR were carried out on an 80 MHz Benchtop NMR spectrometer (Magritek Spinsolve 80 Ultra) at 26 °C. 128 scans were acquired using a pulse angle of 90°, an acquisition time of 6.4 s and a relaxation delay of 15 s.

Reaction of crude TPA with EG to produce cyclics and oligomers

A stock solution of EG, (35.8 mmol) and catalyst ([Catalyst]:[EG] = 0.0005) was prepared in a 4 ml vial. The required amount of TPA was weighed into 20 ml vials equipped with lids (polypropylene with PTFE septa) pierced by Pasteur pipettes, functioning as condensers. A stirring bar was added into each vial together with 0.34 mL of the prepared stock solution per each 0.5 g of TPA ([TPA]:[EG]:[Catalyst] = 1:2:0.001 assuming 100% pure TPA). The mixtures were first heated to 150 °C for 2 hours under constant stirring, to get a homogenous paste. Temperature was then increased to 200 °C. The reaction was stopped after the times specified in the discussion and analysed using Q-NMR. Extraction was performed by adding 10 mL of anisole into the vials followed by heating to 150 °C under constant stirring for 1 hour. The hot mixture was then filtered using a paper filter, and the liquid part was collected in a tared 20 mL vial. The residual, insoluble solid on the filter and the reaction vial were additionally washed with hot anisole to optimise the amount of soluble material extracted. The combined anisole fractions were evaporated using a rotary evaporator at reduced pressure and a water bath temperature not exceeding 50 °C. The vial was then placed in a vacuum oven (approximately 5 mbar) at room temperature overnight to remove residual solvent. The resulting mixture was weighed to determine the yield and analysed by Q-NMR.

Polymerization of the purified mixed cyclics and BHET

A simultaneous condensation polymerization and ring opening polymerisation (ROP) was performed by placing 100 mg of the direct product (containing BHET, cyclics and possibly short linear oligomers as discussed in the text and residual catalyst) into a 4 mL glass vial together with a magnetic stirring bar. The reaction was carried out at 280 °C for 15 minutes under a constant nitrogen flow. A sample of the cooled reaction mixture was transferred to an NMR tube and dissolved in a mixture of deuterated chloroform and trifluoroacetic acid (TFA) (4:1 v/v).

The obtained product could be chain extended by Solid State Polymerisation (SSP)

through the functional end-groups that are always present for a condensation product. The following procedure was used: the 4 mL vial with the remaining product from the ROP was placed in a vacuum oven at 5 mbar overnight at 200 °C. As before, a sample was analysed by ^{1}H-NMR in a deuterated chloroform:TFA mixture (4:1).

Size Exclusion Chromatography

Size exclusion chromatography was carried out on a chromatographic system consisting of a Waters Acquity solvent delivery module and column oven, connected to a Waters PDA TS detector. The columns were a 150 x 4.6 mm Acquity APC™ XT 200 2.5 µm and a 150 x 4.6 mm Acquity APC™ XT 125 x 2.5 µm organic size exclusion chromatography columns connected in series; this column combination provides resolution from approximately 1000 to 70000 Daltons. The eluent was a mixture of dichloromethane and hexafluoroisopropanol (9:1 v/v) and the absorption signal at 290 nm (using a slit width of 12 nm) was used for detection. PET samples were dissolved in the eluent overnight and filtered through a 0.45 µm PTFE filter prior to injection.

Microscopy

The products from solid state polymerization were placed on a microscope slide. The glass was then heated above melting temperature of the synthetized polymers. The coverslip was then placed on top of the slider to press the sample.
Microscope images were acquired using a KEYENCE VHX-7000 Digital Microscope. A white stage plate was used as a background for all the pictures. The pictures were captured using reflected non-polarized light at a magnification of 20X. To ensure consistency, all the pictures were taken using the same brightness settings.

Table S3. cPET weight loss and released monomers measured through HPLC.

Sample	Weight loss [%]	Released monomer [mM]
**PET2 0.5 x 3.0 cm**	0	0
**PET2 10 x 3.0 cm**	0	0

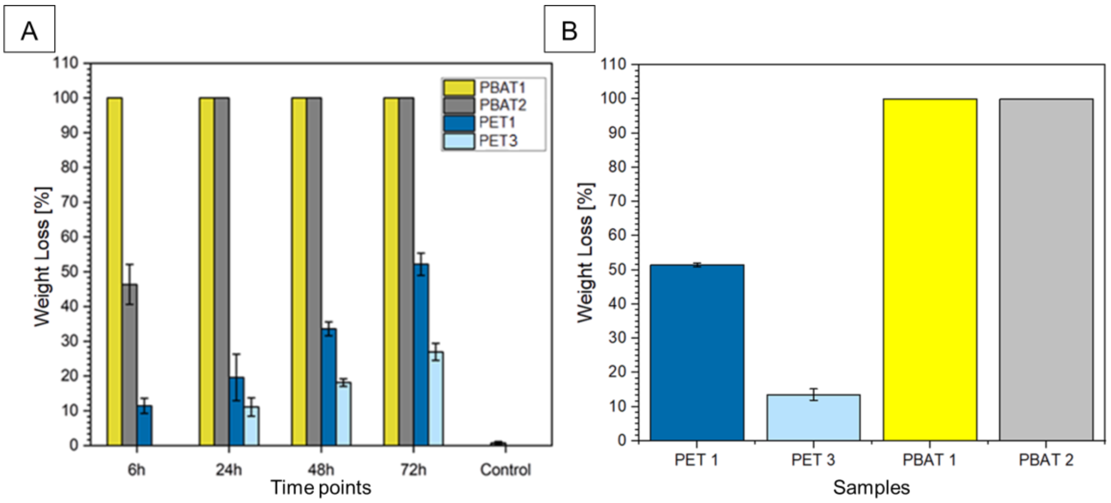

Figure S2. Enzymatic hydrolysis of aromatic-aliphatic polyesters. A: Small scale enzymatic hydrolysis. Time course weight loss of 0.5 cm x 3 cm treated films compared to control reactions (buffer solution). Treated samples were PBAT1 (yellow bars), PBAT2 (grey bars), PET1 (blue bars), PET3 (light blue bars). B: Weight loss of 10 cm x 3 cm treated films after 72 h reaction. Treated samples: PET1 (blue bars), PET3 (light blue bars), PBAT2 (grey bars), PBAT1 (yellow bars). The error bars represent the average of triplicate measurements. No standard deviation bar for PBAT samples, due to a 100% weight loss in all the triplicates.

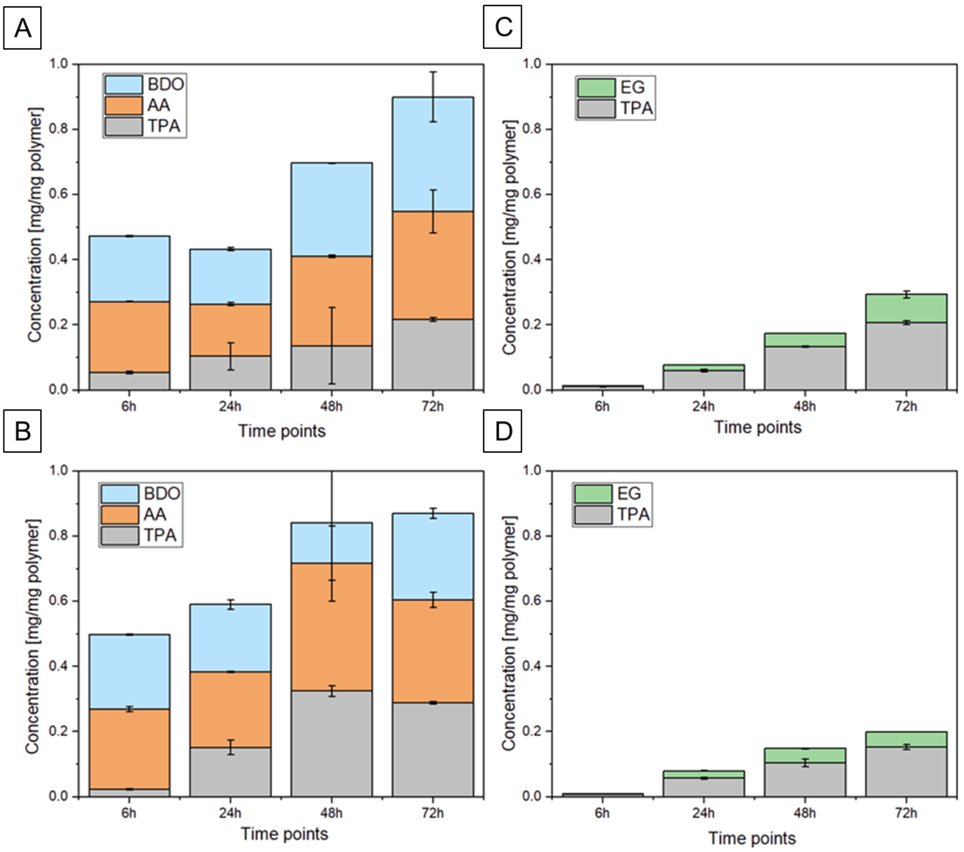

Figure S3. HPLC analysis of released soluble monomers from 0.5 cm x 3 cm films. A: PBAT1; B: PBAT2, C: PET1; D: PET3. Grey bars: terephthalic acid (TPA); orange bars: adipic acid (AA); light blue bars: 1,4-butanediol (BDO); green bars: ethylene glycol (EG). The represented data are the average of triplicate measurements.

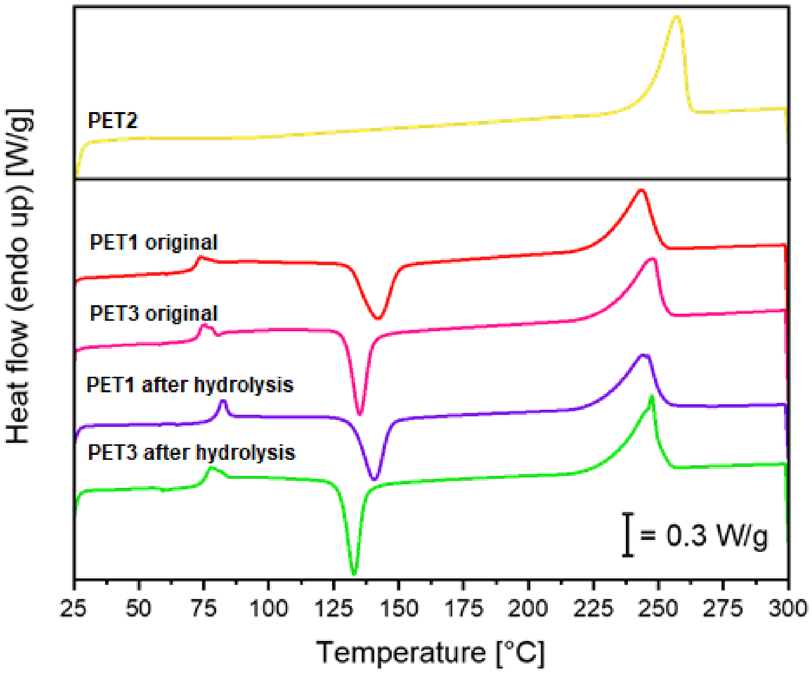

Figure S4. DSC traces of PET2 before hydrolysis (yellow) and PET1 and PET3 before (red and pink, respectively) and after hydrolysis (violet and green, respectively).

Table S4. DSC characterization of PET samples before and after enzymatic hydrolysis.

Sample	Tg [°C]	Tcc [°C]	ΔHcc [J/g]	Tm [°C]	ΔHm [J/g]	χc [%]
**PET1**	71	142	-40	243	39	0
**PET2**	90	-	-	257	49	35
**PET3**	72	135	-38	248	37	0
**PET1-residual**	77	141	-35	244	37	1
**PET3-residual**	74	133	-40	247	39	0

*:The subscript cc and m indicate the values measured during cold crystallization and melting processes, respectively.

Table S5. Comparison of weight losses of the same polymer incubated with different concentration of HiC

Sample	Initial Weight (mg)	HiC concentration (μM)	Weight loss (%)
**PET1 10 cm x 3 cm**	454.39	5	50
	450.65	7.5	72
	443.09	10	96
	441.97	15	96

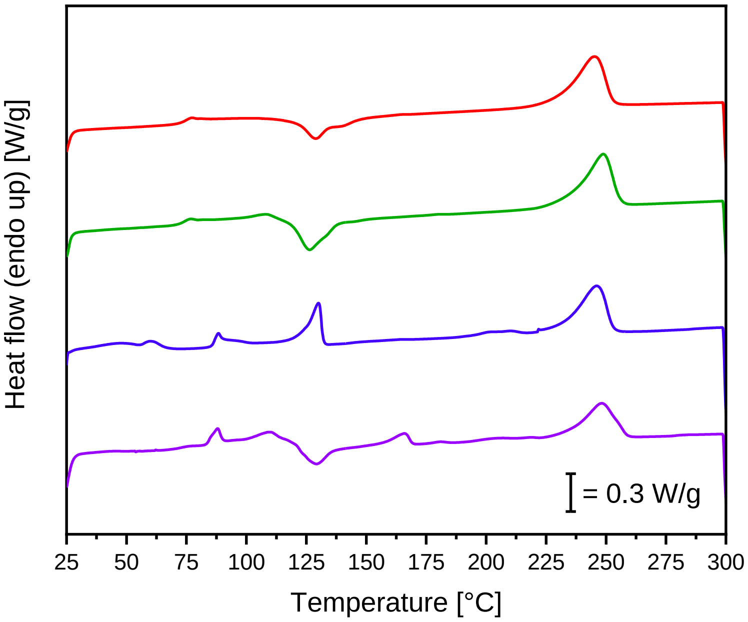

Figure S5. Differential Scanning Calorimetry of PW1 and PW2 before (red and green, respectively) and after hydrolysis (blue and violet, respectively).

Table S6. DSC characterization of PET waste samples before and after enzymatic hydrolysis.

Sample	T_g [°C]	T_{cc} [°C]	ΔH_{cc} [J/g]	T_m [°C]	ΔH_m [J/g]	χ_c [%]
**PW1**	73	129	-19	245	43	17
**PW2**	72	126	-24	249	39	11
**PW1-residual**	85	-	-	246	31	22
**PW2-residual**	83	129	-14	248	30	11

The subscript “cc” and “m” indicate the values measured during cold crystallization and melting processes, respectively

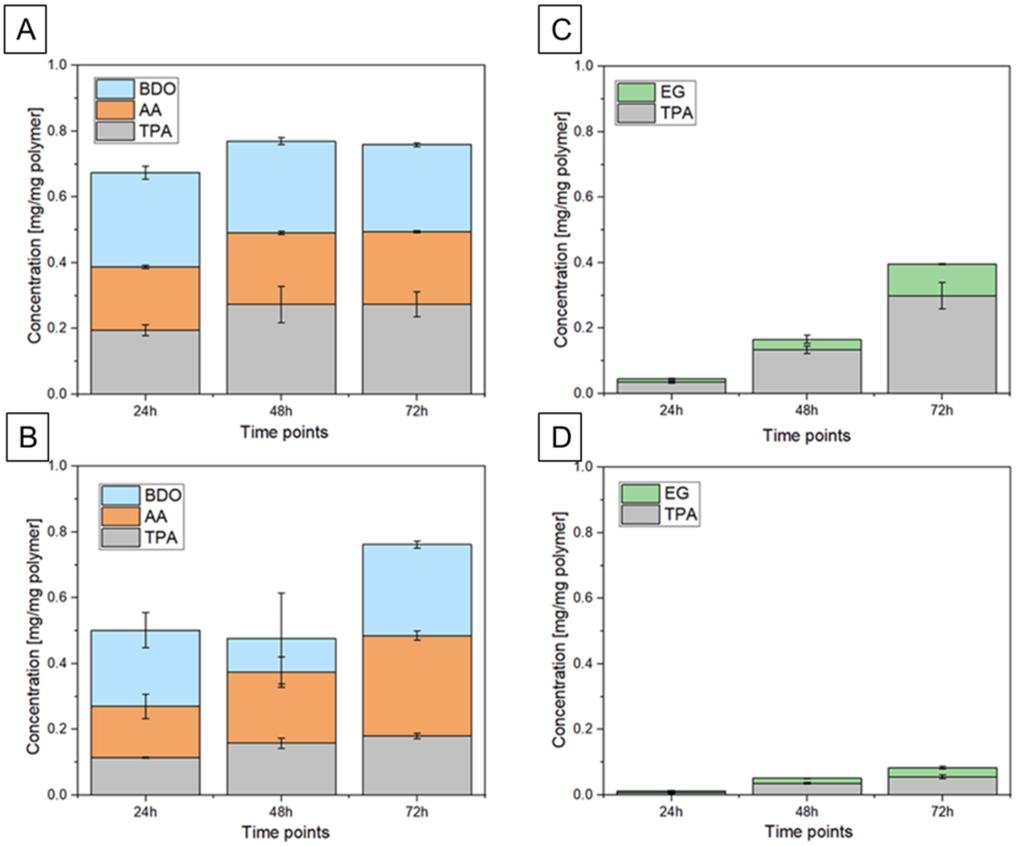

Figure S6. HPLC analysis of soluble monomers release by the enzyme HiC from 10 cm x 3 cm films. A: PBAT1; B: PBAT2, C: PET1; D: PET3. Grey bars: terephthalic acid (TPA); orange bars: adipic acid (AA); light blue bars: 1,4 butanediol (BDO); green bars: ethylene glycol (EG). The represented data are the average of triplicate measurements.

Surface characterization polyesters after partial hydrolysis

Samples after partial hydrolysis were characterized through ATR FT-IR.

In PET films particularly, a decrease of relative absorbance was recorded at 1710 cm^{-1}, 1240 cm^{-1}, and 1090 cm^{-1}, corresponding respectively to C=O carbonyl stretching, C-O stretching of ester group and vibrations of ester C-O bond (Figure S7-S10). Moreover, the latter peak was slightly different in terms of shape. The region between 1340 cm^{-1} and 1410 cm^{-1} includes peaks that are attributed to the trans conformation of EG of crystalline PET, namely 1341 cm^{-1} (CH_2 wagging), while 1410 cm^{-1} is considered as an indication of the ring C-H in plane bending, as well as C-C stretching. Since these two bands are characteristic of either the crystalline or amorphous form of PET, their ratio can be used as a reference value for crystallinity. In particular, calculating the relative absorbance of the normalized spectra at 1341 cm^{-1} divided by the absorbance at 1410 cm^{-1}, could be done for all the spectra of both PET samples, giving the outputs specified in Table S7 (and shown in Figures S7-S10)[31]. As the value

increases from the control/blank sample to the 72 h incubated samples, this indicates an increase of crystallinity in the residual PET.

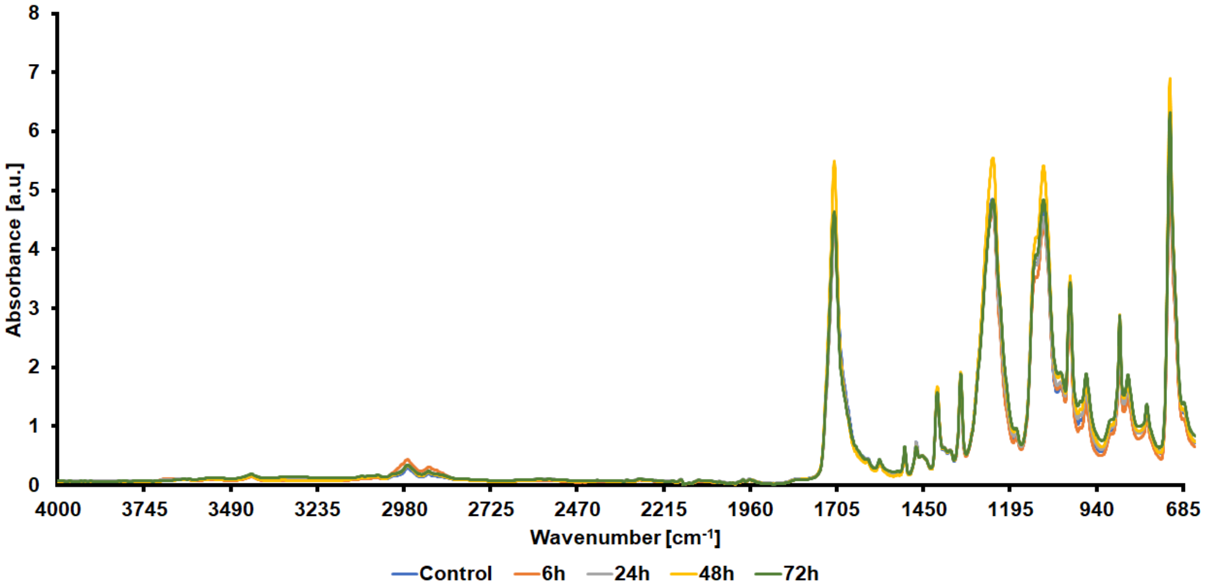

Figure S7. FT-IR ATR spectra between 4000 cm^{-1} to 685 cm^{-1} of residual PET2 films (0.5 cm x 3 cm) after incubation with enzyme. Blue line: control (no enzyme); orange line: 6 h; grey line: 24 h; yellow line:48 h; light blue line:72 h.

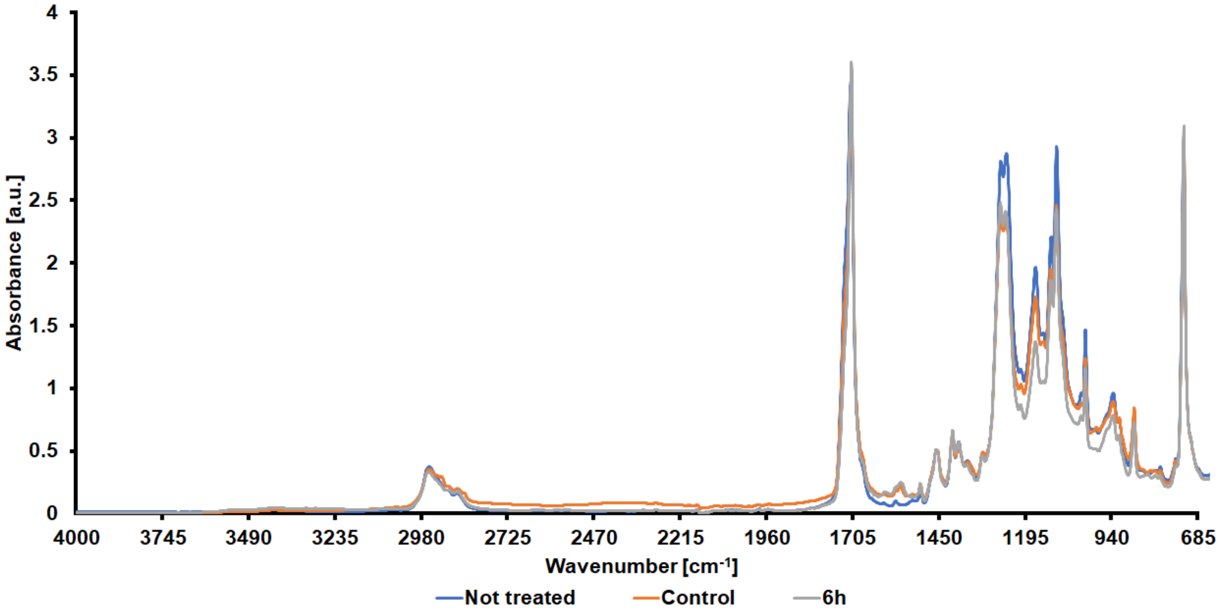

Figure S8. FT-IR ATR spectra between 4000 cm^{-1} to 685 cm^{-1} of residual PBAT2 films (0.5 cm x 3 cm) after incubation with enzyme. Blue line: not treated (original polymer); orange line: control (no enzyme); grey line: 24 h; yellow line: 48 h; light blue line: 72 h.

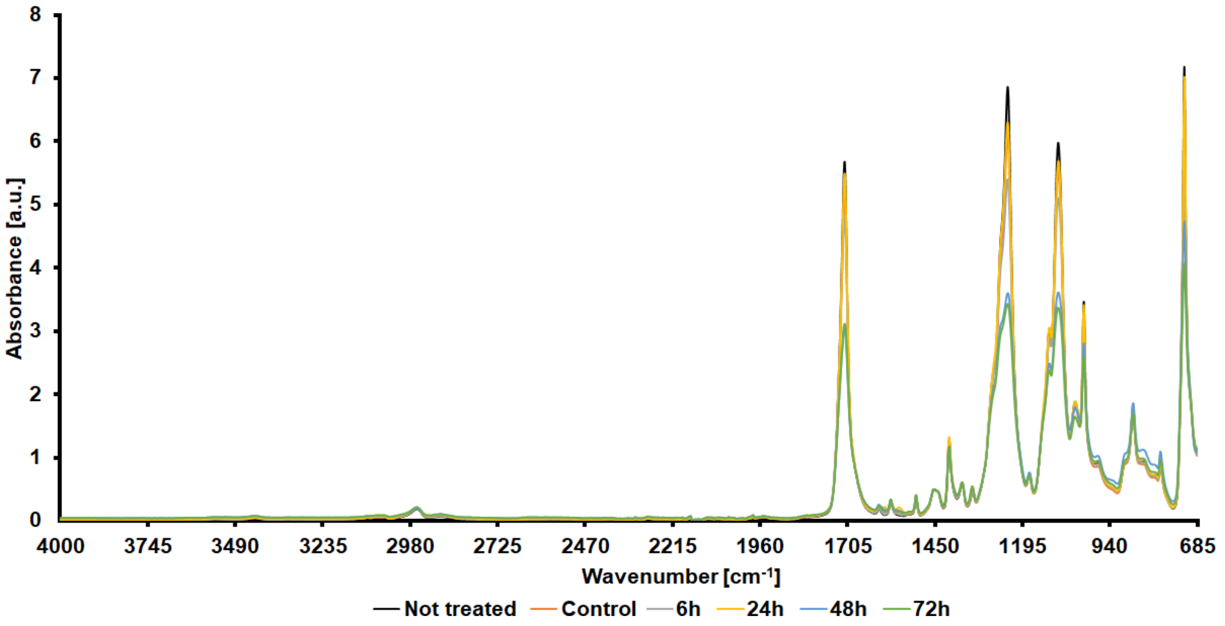

Figure S9. FT-IR ATR spectra between 4000 cm^{-1} to 685 cm^{-1} of residual PET1 films (0.5 cm x 3 cm) after incubation with enzyme. Black line: not treated (original polymer); orange line: control (no enzyme); grey line: 6 h; yellow line: 24 h; light blue line: 48 h; green line: 72 h

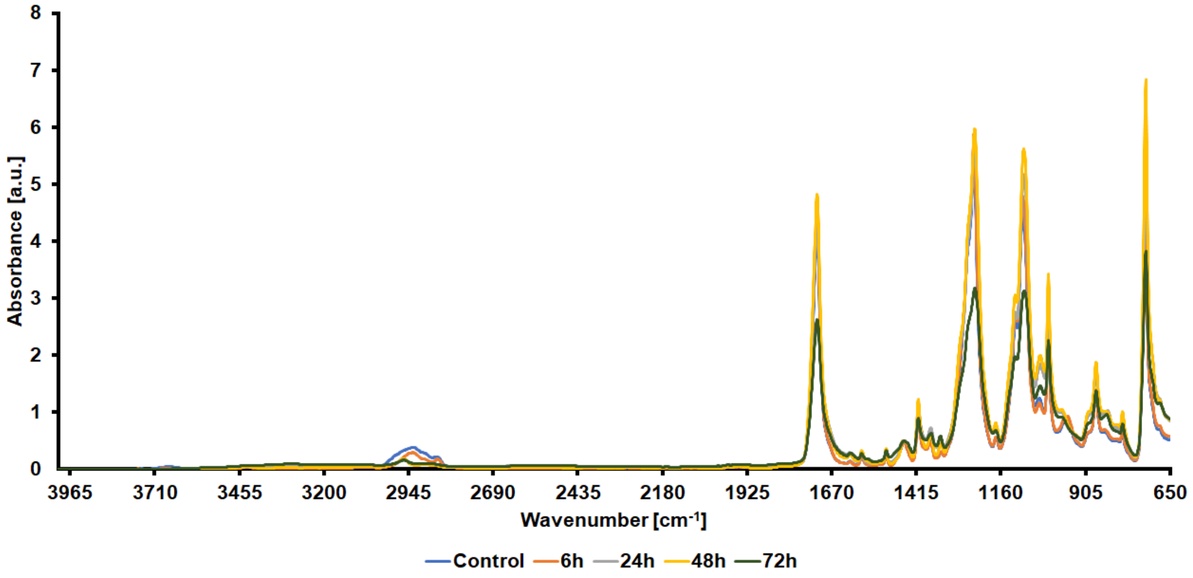

Figure S10. FT-IR ATR spectra between 4000 cm^{-1} to 685 cm^{-1} of residual PET3 films (0.5 cm x 3 cm) after incubation with enzyme. Blue line: not treated (original polymer); orange line: 6h; grey line: 24 h; yellow line: 48 h; light blue line: 72 h

Table S7. Relative absorbance of each FT-IR ATR spectrum at 1341 cm^{-1} and 1410 cm^{-1} and the corresponding ratio for crystallinity estimation

PET3	A 1341	A 1410	A 1341/A 1410
**Blank**	0.31	0.99	0.31
**Control**	0.3	0.99	0.30
**6 hs**	0.31	0.99	0.31
**24 hs**	0.56	1.19	0.47
**48 hs**	0.9	1.24	0.73
**72 hs**	0.59	0.9	0.66
**PET1**	**A 1341**	**A 1410**	**A 1341/A 1410**
**Blank**	0.48	1.28	0.38
**Control**	0.46	1.24	0.37
**6 hs**	0.47	1.26	0.37
**24 hs**	0.55	1.33	0.41
**48 hs**	0.53	1.18	0.45

Analysis of recovered materials: FT-IR, ^{1}H-NMR

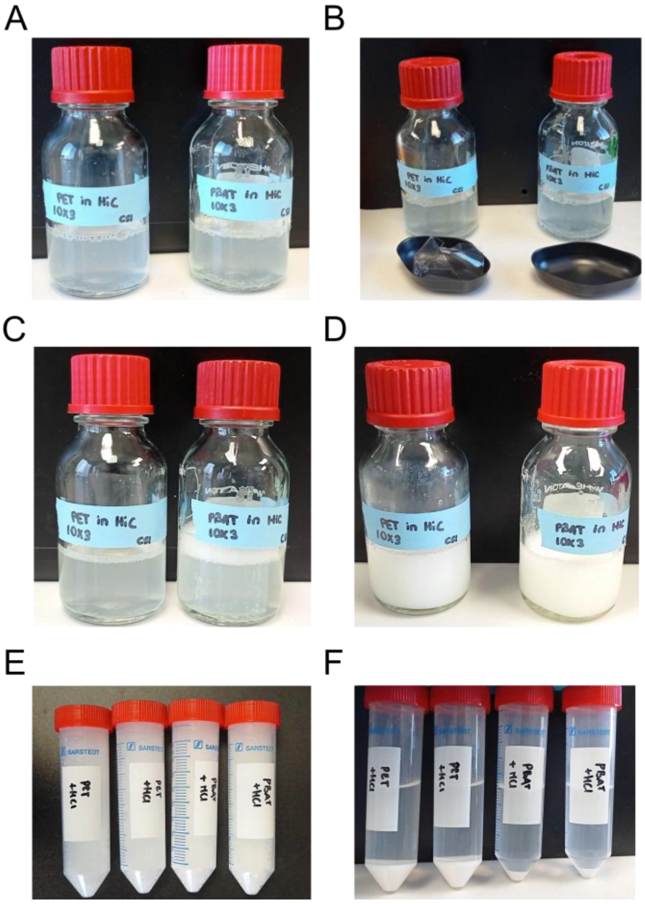

Figure S11. Recovery of TPA from PET and PBAT samples. A: Hydrolysis time zero; B: Hydrolysate after 72 hours (end reaction); C: hydrolysates without residual films; D: acidified hydrolysates for TPA recovery; E: acidified solutions transferred in falcons for centrifugation; F: falcons after centrifugation and precipitation of TPA as a white pellet. No further phase separations in the remaining liquid.

Table S8. Monomer amount [mM] in PET3 and PBAT2 before and after the precipitation of terephthalic acid by means of acidification

PET3	Terephthalic acid [mM]	Ethylene Glycol [mM]	
**Final concentration**	16.33±0.94	30.08±0.23	
**After HCl**	0.00	29.19±0.39	
**PBAT2**	**Terephthalic acid [mm]**	**Adipic acid [mM]**	**1,4-butanediol [mM]**
**Final concentration**	7.93±0.17	14.70±0.05	24.13±0.49
**After HCl**	0.00	14.00±0.27	23.63±0.04

FT-IR (see Figure S12-S17) provided the chemical fingerprint of the recovered materials, whose similarity with the pure TPA was taken as an indication of the high purity level. The final step of acidification led in all four cases, to a spectrum that is superimposable to pure commercial TPA. Normalization was set between 2470 and 2215 cm^{-1}. The highest analogies were observed for PBAT samples, notably after the second wash in acidic solution. The result can be partially explained considering an overall minor amount of starting material recovered from PBAT hydrolysis solution, therefore a better acidification and wash performance. The characteristic peaks of TPA can be recognised in all the samples and associated to the chemical groups present in the molecule: a broad absorption region between 2500 and 3400 cm^{-1}, where the -OH stretching of dimers and monomers give their signals. The acid bond C=O is visible at a wavenumber around 1670 cm^{-1}, with a shoulder at 1630 cm^{-1}. Signals at 1440 cm^{-1} and 1395 cm^{-1} are instead C-O stretching and C-OH bending associated, while the C-C stretching and C-CH C-C-C bending of benzene ring are coupled and responsive to 814-850 cm^{-1} region. The spectra of the TPA recovered from mixed waste are reported in Figure S16-S17 and displays high similarities to the pure TPA.

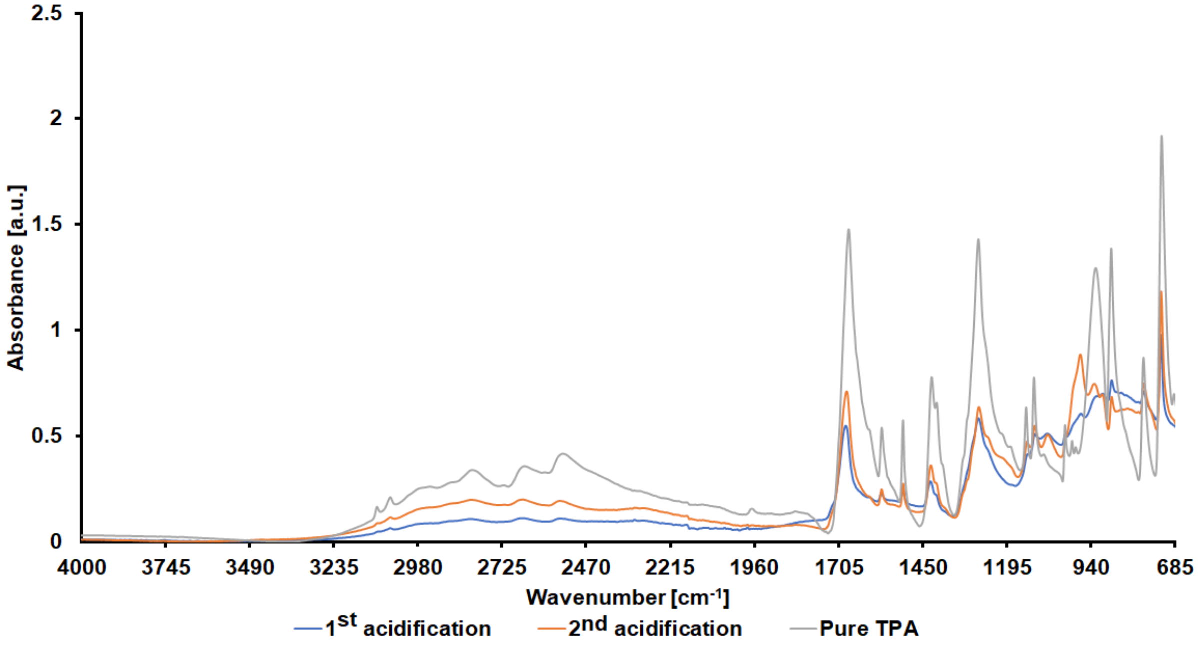

Figure S12. FT-IR ATR not normalized spectra of recovered TPA from hydrolysed 10 x 3 cm PET1 after two acidification steps (1st acidification: blue line; 2nd acidification: orange line) compared to the pure TPA (grey line)

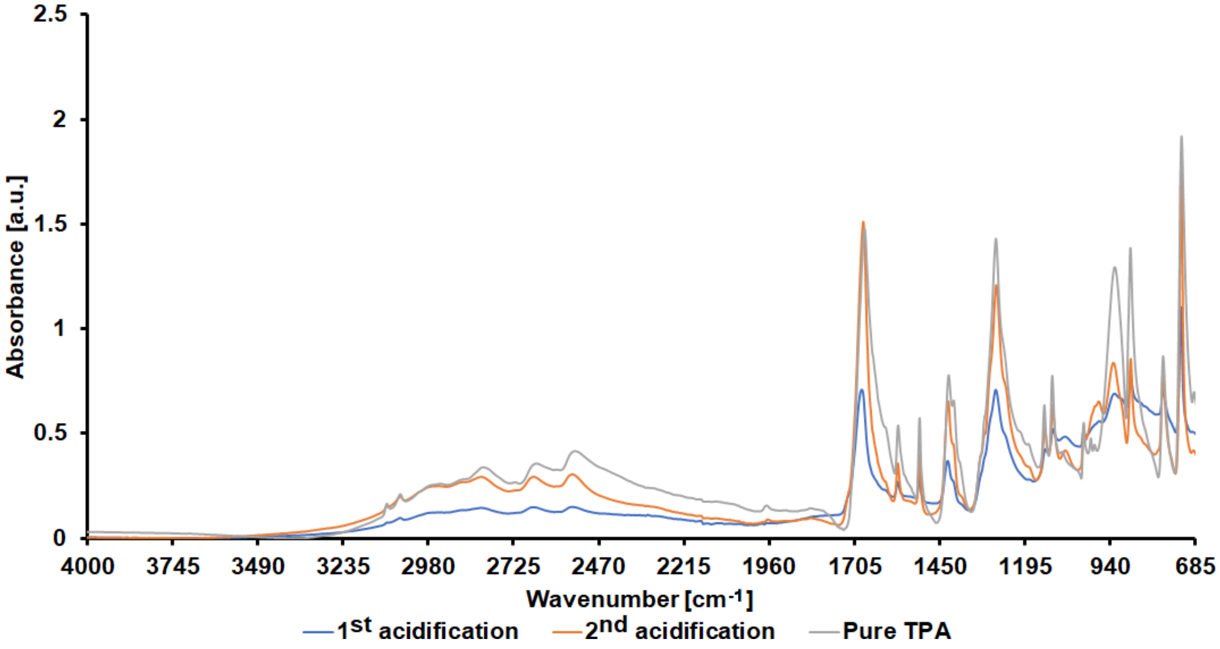

Figure S13. FT-IR ATR not normalized spectra of recovered TPA from hydrolysed 10 x 3 cm PET3 after two acidification steps (1st acidification: blue line; 2nd acidification: orange line) compared to the pure TPA (grey line)

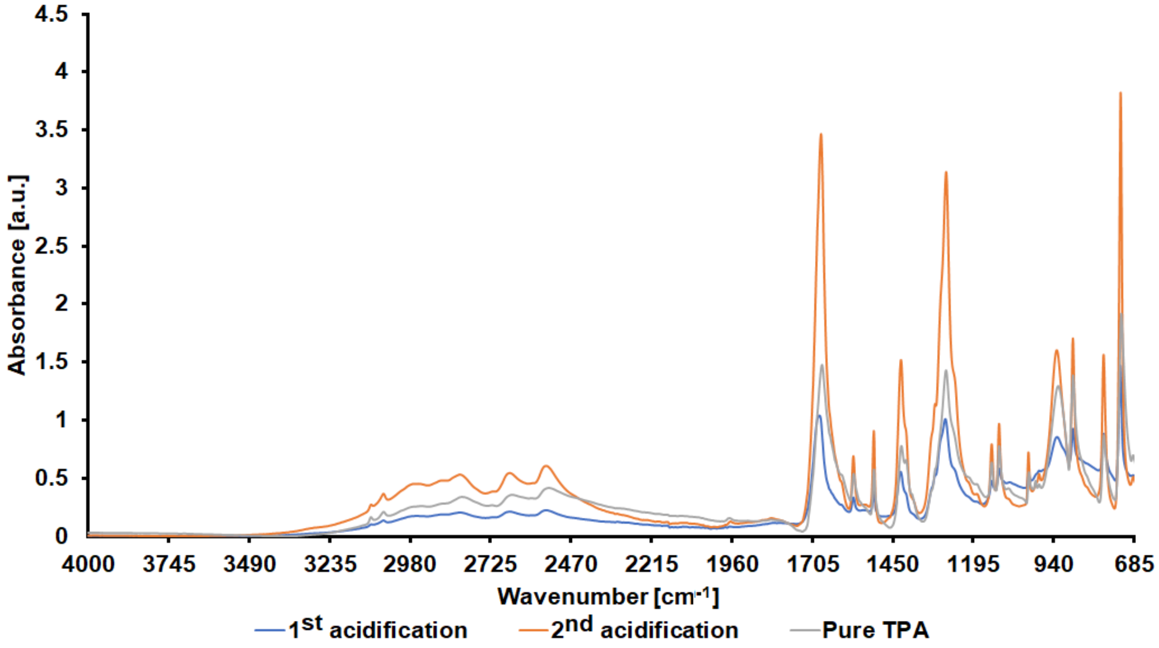

Figure S14. FT-IR ATR not normalized spectra of recovered TPA from hydrolysed 10 x 3 cm PBAT1 after two acidification steps (1st acidification: blue line; 2nd acidification: orange line) compared to the pure TPA (grey line)

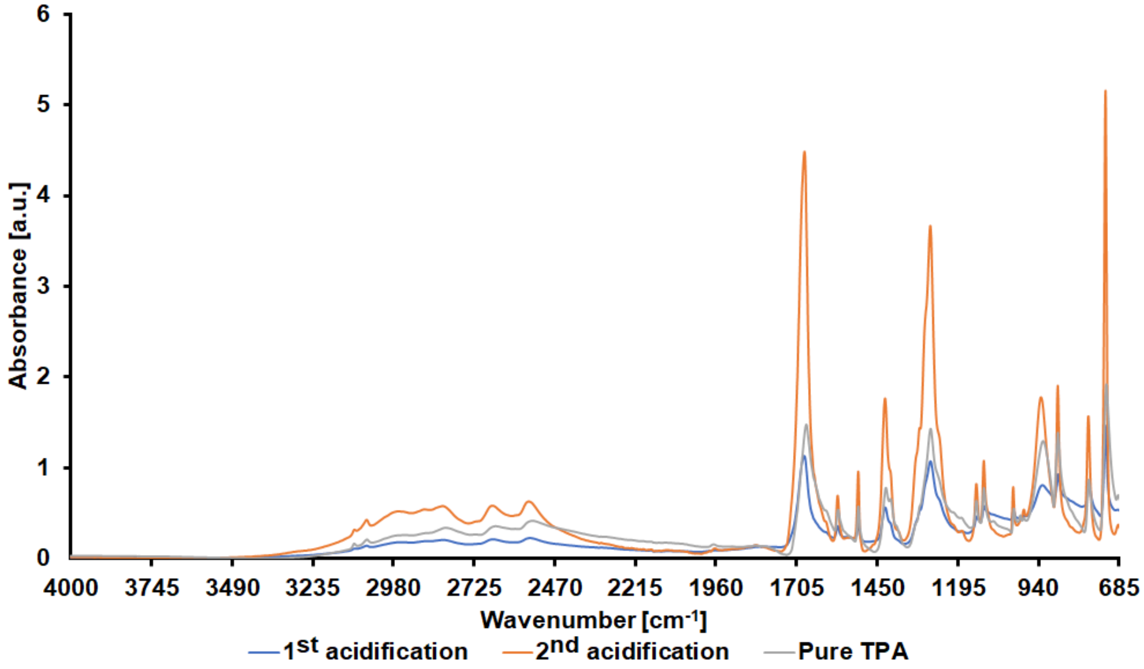

Figure S15. FT-IR ATR not normalized spectra of recovered TPA from hydrolysed 10 x 3 cm PBAT2 after two acidification steps (1st acidification: blue line; 2nd acidification: orange line) compared to the pure TPA (grey line)

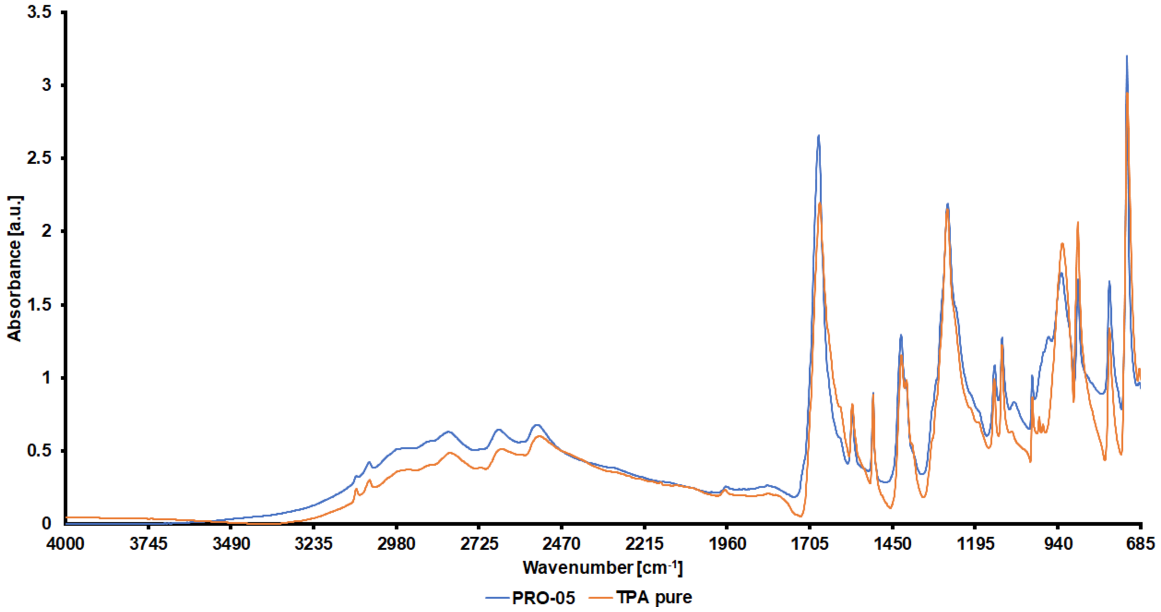

Figure S16. FT-IR ATR spectra of recovered TPA after acidification of PW2 hydrolysate (blue line) compared to the pure TPA (orange line). Normalization between 2470 and 2215 cm^{-1}.

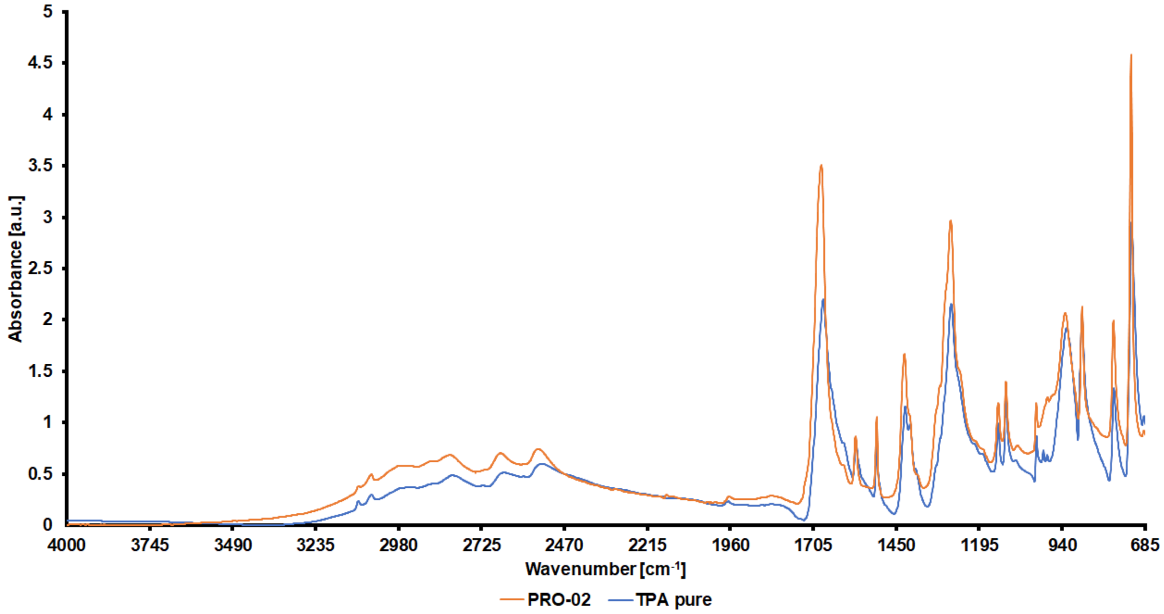

Figure S17. FT-IR ATR spectra of recovered TPA after acidification of PW1 hydrolysate (orange line) compared to the pure TPA (blue line). Normalization between 2470 and 2215 cm^{-1}.

^{1}H-NMR analysis was also carried out to determine the presence of impurities in the recovered TPA as shown in Figure 3. The signal at 8 ppm corresponds to the TPA aromatic ring protons, while in the surrounding region, the ester aromatic proton signals can be detected as low intensity peaks (8.1 ppm). These are present in TPA purified from both PBAT and PET. Other residues are detectable, even though with low intensity. The signals at 3.7 and 4.3 ppm are in fact associated respectively to the protons of EG attached to the aromatic ring (C$\underline{H_2}$-OC=O) and to C$\underline{H_2}$-OH external EG. In all cases the relative amounts of esters were less than 15% based on the total

aromatics, bearing in mind that e.g. co-precipitated salts will typically not be visible in ^{1}H-NMR due to their lack of protons. Similarly, terephthalic acid and any residual potassium terephthalates will be indistinguishable due to fast exchange.

As concerns TPA obtained from PET3 or PET1 (Figure S18, Table S9), had a relative acid content of 92% and 88% respectively. Similar numbers were found for the TPA isolated from PBAT (Figure S19, Table S10), in which the esters accounted for less than 10% (ranging from 90 to 96% for PBAT1 and PBAT2 respectively). A comparative level was also found for the TPA recovered from PW1 and PW2 samples, as confirmed by FT-IR (Spectra in Figure S16-17) and ^{1}H-NMR (purity 99% and 84% respectively; Figures S20 and Table S11).

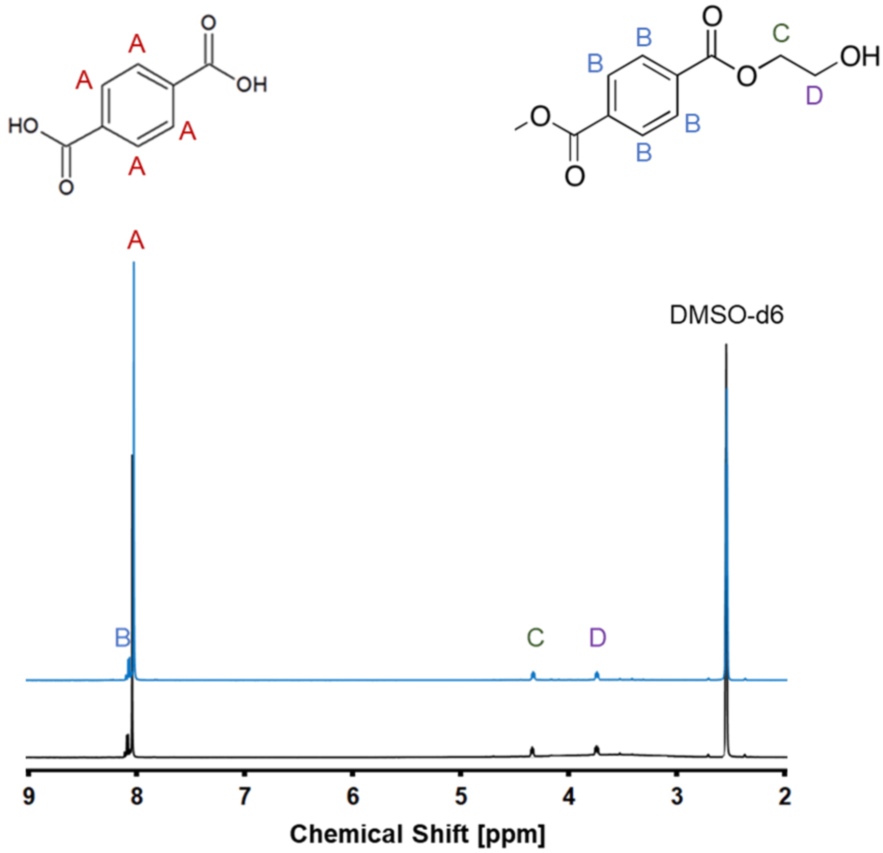

Figure S18. ^{1}H-NMR spectra of TPA recovered from PET3 (blue) and PET1 (black). Integral values for all signals are available in ESI, Table S5.

Table S9. Integrated area PET3 and PET1

Ppm	Assignment	Protons	Integrated Area PET3	Integrated Area PET1
**8**	TA	4	1	1
**4.3**	EG	4	0.33	0.54
**3.67**	EG	4	0.34	0.80
**8.10**	Oligomers	4	-	0.22
**Purity [%]**			92	88

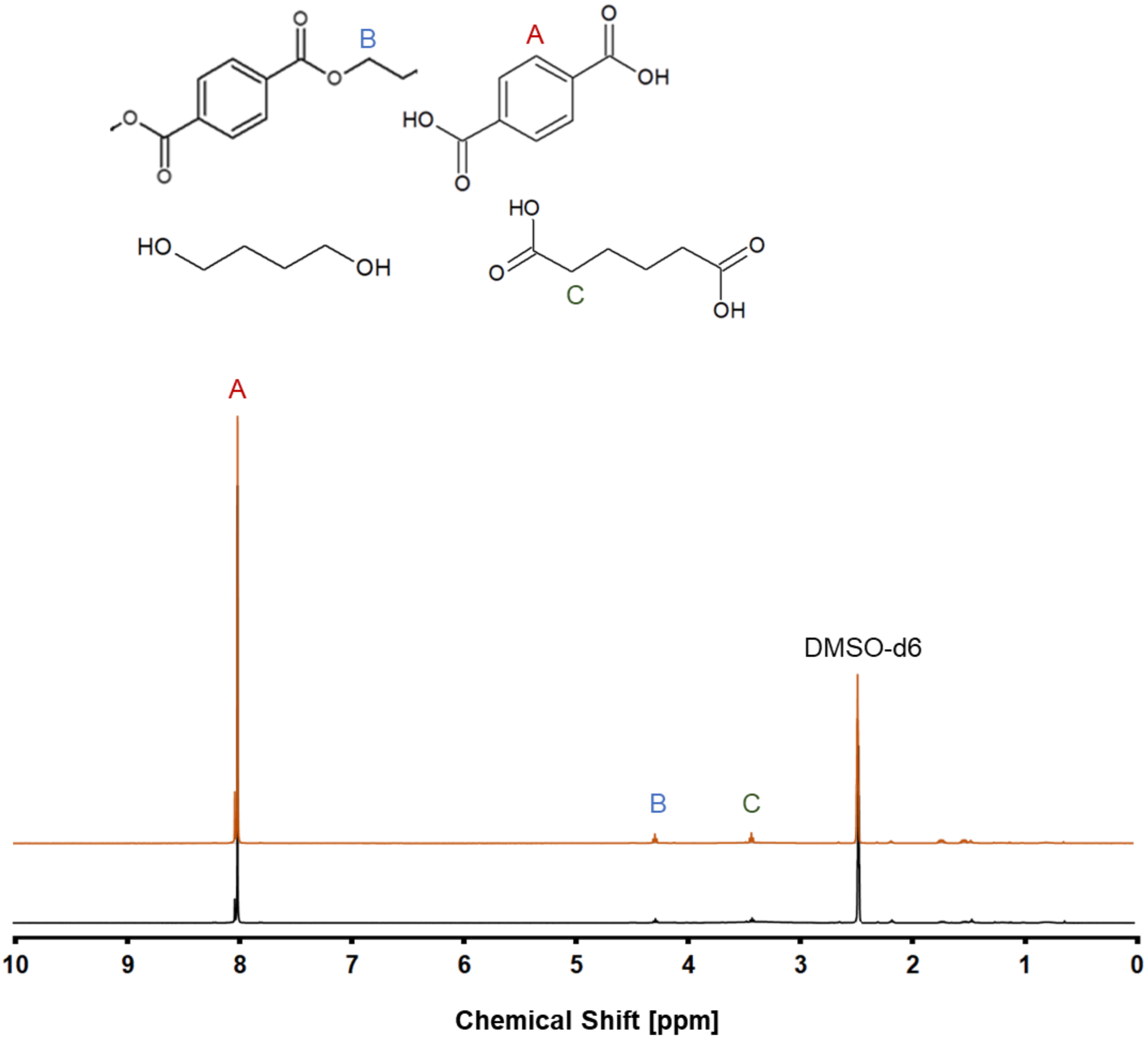

Figure S19. ^{1}H-NMR of TPA obtained from PBAT1 (Black) and PBAT2 (Orange).

Table S10. Integrated Area PBAT1 and PBAT2

Ppm	Assignment	Protons	Integrated Area PBAT2	Integrated Area PBAT1
**8**	TA	4	1	1
**4.3**	AA	4	0.26	0.17
**1.72**	Internal CH2	4	0.24	
**8.10**	Oligomers	4	0.43	
**Purity [%]**			90	96

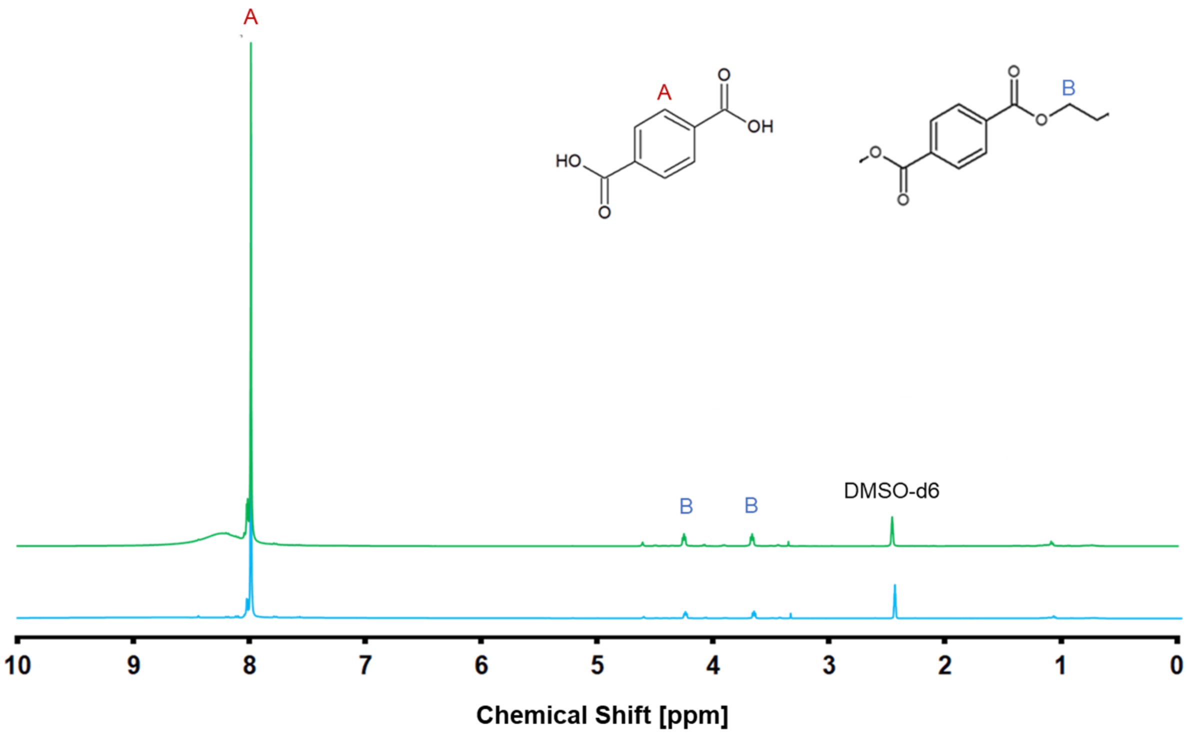

Figure S20. Comparison between ^{1}H-NMR spectra of TPA recovered from sample PW1 (Light blue) and PW2 (Green)

Table S11. Integrated Area PW1 and PW2

Ppm	Assignment	Protons	Integrated Area PW1	Integrated Area PW2
**8**	TA	4	1	1
**4.3**	EG	4	0.03	0.05
**3.67**	EG	4	0.03	0.05
**8.10**	Oligomers	4	-	0.74
**Purity [%]**			99	84

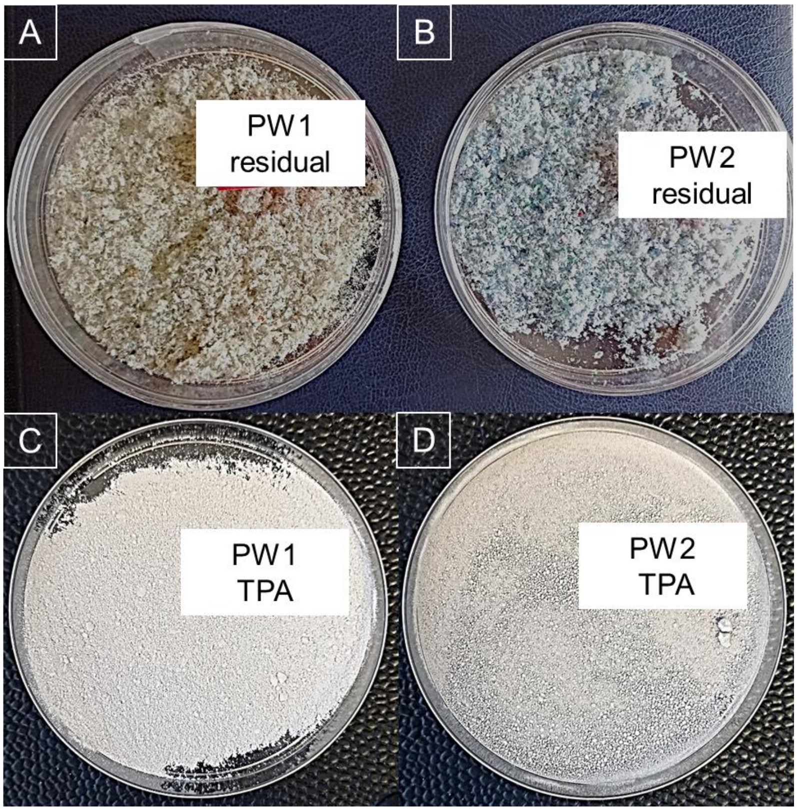

Figure S21. PW1 and PW2 residual after hydrolysis and the TPA derived respectively from each hydrolysate. A: residual not hydrolysed material from PW1; B: residual not hydrolysed material from PW2; C: recovered TPA from PW1 hydrolysate; D: recovered TPA from PW2 hydrolysate

Table S12. TGA characterization of TPA samples recovered after enzymatic hydrolysis.

Sample	T_{onset} [°C]	T_{max} [°C]	Residue at 800 °C [%]
Pure TPA	374	400	0.0
PET1	339	367	2.9
PET3	331	354	0.9
PBAT1	323	356	11.7
PBAT2	298	330	5.6
PW1	320	351	7.4
PW2	314	342	25.9

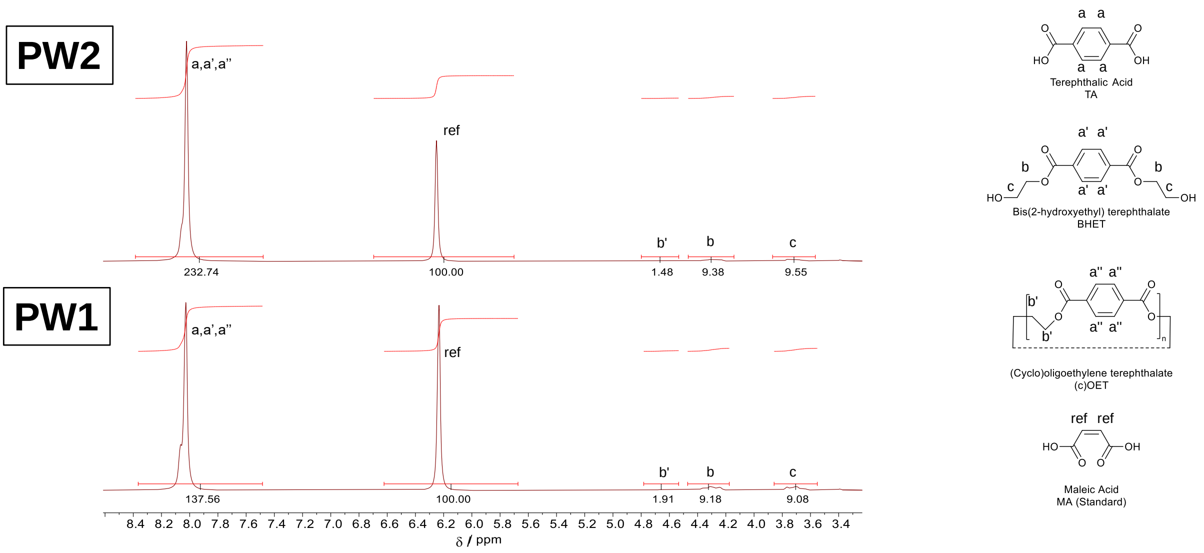

Figure S22. Assigned ^{1}H-NMR spectra of recovered TPA containing Maleic Acid standard.

Table S13. Q-NMR of recovered TPA

Sample	TPA content / % w/w
**PW1**	60
**PW2**	85

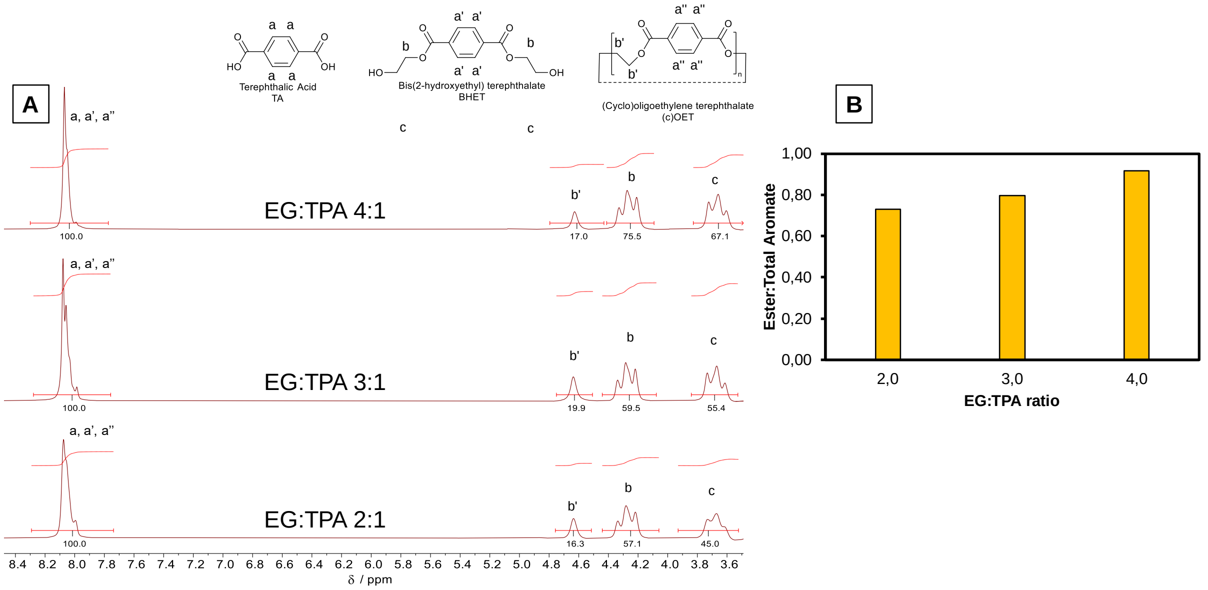

Figure S23. Reaction of ethylene glycol and terephthalic acid at different molar ratios. A: ^{1}H NMR spectra at different ratios. B: Total amount of ester (sum of integrals b+b') divided by total aromate (integrals a, a', a''). Reaction conditions: 1) 150 °C, 2 h 2) 200 °C, 3 h. Catalyst: Titanium tetrabutoxide (TPA:Cat = 1000:1 mol:mol)

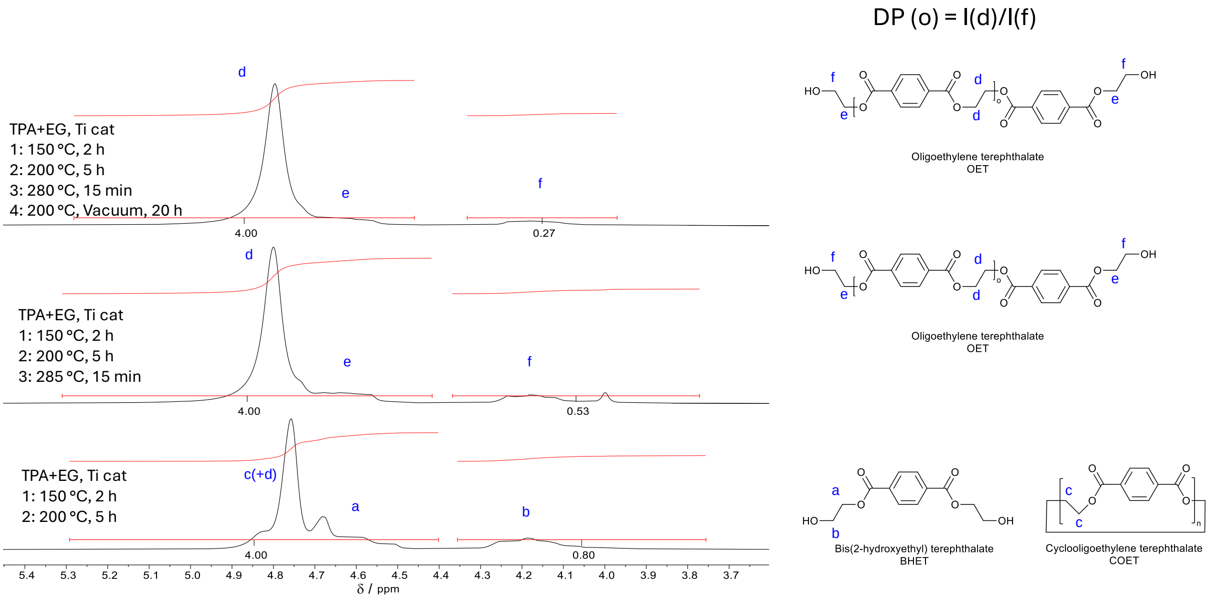

Figure S24. Assigned ^{1}H NMR spectra after initial esterification, after ring-opening polymerisation and after solid-state polymerisation. Solvent: $CDCl_3$:TFA 3:1

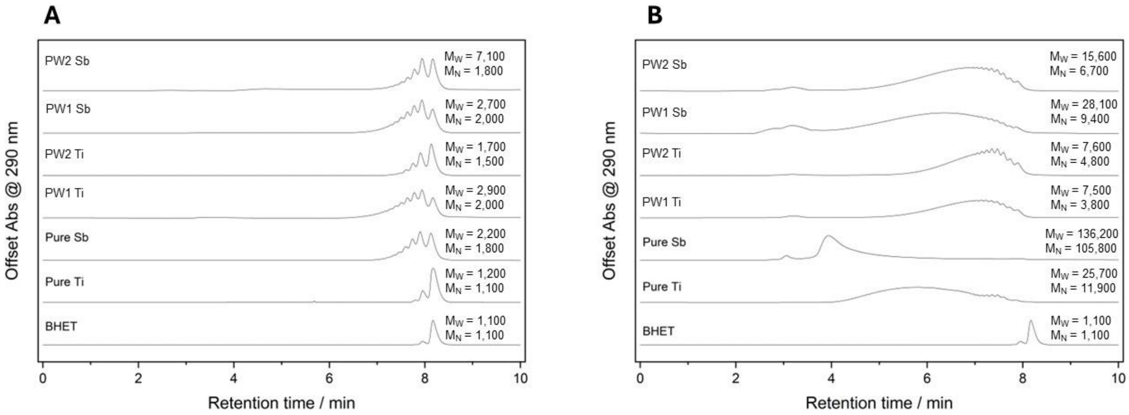

Figure S25. Size exclusion chromatography of reactions between terephthalic acid (pure and recovered) and ethylene glycol A) after 2 h reaction at 150 °C followed by 5 h reaction at 200 °C. B) after extraction of reaction mixture from A with anisole, followed by drying, treatment at 280 °C for 15 min and 12 h at 200 °C, 5 mbar. Chromatography in CH_2Cl_2:HFIP 9:1, molecular weights calculated against polystyrene standards.

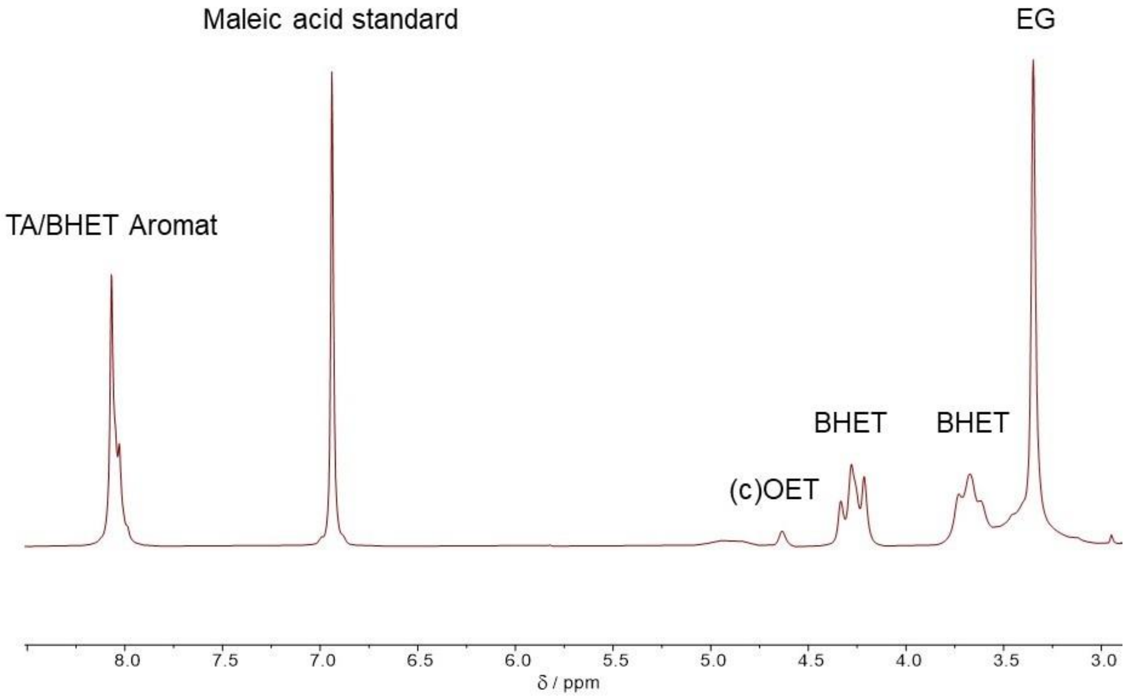

Figure S26. ^{1}H Q NMR of reaction mixture (5 h Ti) after extraction with anisole.

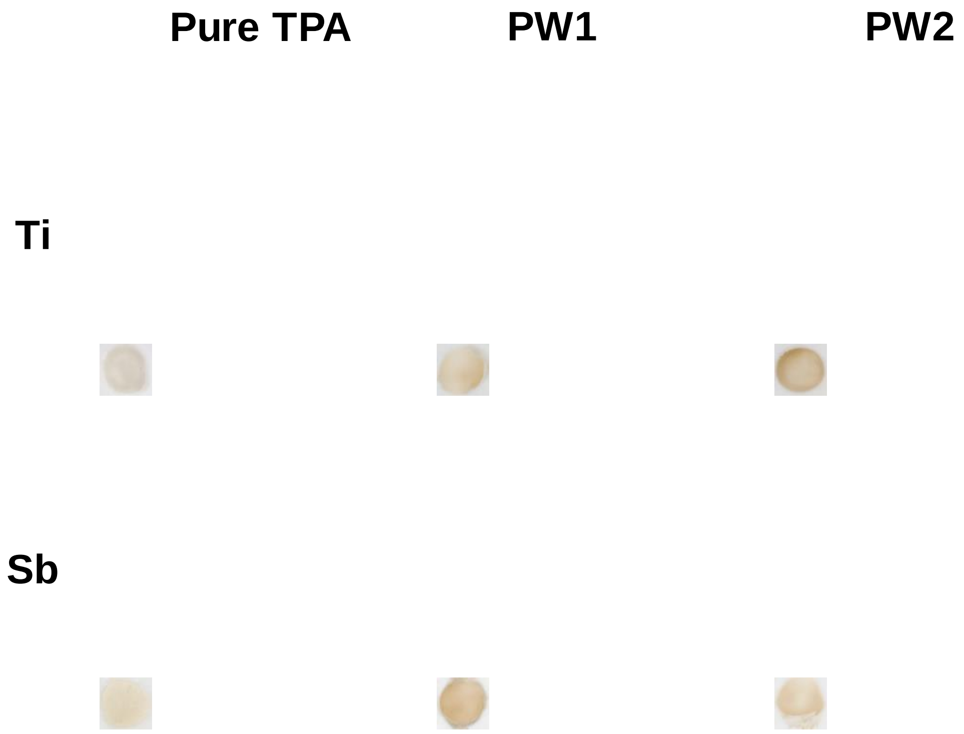

Figure S27. Microscopy images of pure and recovered TPA after reaction with ethylene glycol and subsequent polymerization

References

1. Siracusa C, Quartinello F, Soccio M, et al. On the Selective Enzymatic Recycling of Poly(pentamethylene 2,5-furanoate)/Poly(lactic acid) Blends and Multiblock Copolymers. *ACS Sustain Chem Eng*. 2023;11(26):9751-9760. doi:10.1021/acssuschemeng.3c01796

2. Bertolini FA, Soccio M, Weinberger S, et al. Unveiling the Enzymatic Degradation Process of Biobased Thiophene Polyesters. *Front Chem*. 2021;9(November):1-13. doi:10.3389/fchem.2021.771612

3. Weinberger S, Canadell J, Quartinello F, et al. Enzymatic degradation of poly(Ethylene 2,5-furanoate) powders and amorphous films. *Catalysts*. 2017;7(11). doi:10.3390/catal7110318

4. EMD Millipore Corporation. 1.10537.0001 - Carrez clarification. 2020;49(1):1.

5. Heidrich D, Gehde M. The 3-Phase Structure of Polyesters (PBT, PET) after Isothermal and Non-Isothermal Crystallization. *Polymers (Basel)*. 2022;14(4). doi:10.3390/polym14040793
